# Cross-kingdom RNA interference mediated by insect salivary microRNAs may suppress plant immunity

**DOI:** 10.1073/pnas.2318783121

**Published:** 2024-04-08

**Authors:** Ze-Long Zhang, Xiao-Jing Wang, Jia-Bao Lu, Hai-Bin Lu, Zhuang-Xin Ye, Zhong-Tian Xu, Chao Zhang, Jian-Ping Chen, Jun-Min Li, Chuan-Xi Zhang, Hai-Jian Huang

**Affiliations:** ^a^State Key Laboratory for Managing Biotic and Chemical Threats to the Quality and Safety of Agro-products, Key Laboratory of Biotechnology in Plant Protection of Ministry of Agriculture and Zhejiang Province, Institute of Plant Virology, Ningbo University, Ningbo 315211, China; ^b^Department of Plant Pathology, College of Plant Protection, Henan Agricultural University, Zhengzhou 450002, China

**Keywords:** cross-kingdom RNAi, insect–plant interaction, saliva

## Abstract

Communication between insects and plants relies on the exchange of bioactive molecules that traverse the species interface. Although proteinic effectors have been extensively studied, our knowledge of other molecules involved in this process remains limited. In this study, we investigate the role of salivary microRNAs (miRNAs) from the rice planthopper *Nilaparvata lugens* in suppressing plant immunity. A total of three miRNAs were confirmed to be secreted into host plants during insect feeding. Notably, the sequence-conserved miR-7-5P is specifically expressed in the salivary glands of *N. lugens* and is secreted into saliva, distinguishing it significantly from homologues found in other insects. Silencing miR-7-5P negatively affects *N. lugens* feeding on rice plants, but not on artificial diets. The impaired feeding performance of miR-7-5P-silenced insects can be rescued by transgenic plants overexpressing miR-7-5P. Through target prediction and experimental testing, we demonstrate that miR-7-5P targets multiple plant genes, including the immune-associated bZIP transcription factor 43 (OsbZIP43). Infestation of rice plants by miR-7-5P-silenced insects leads to the increased expression of *OsbZIP43*, while the presence of miR-7-5P counteracts this upregulation effect. Furthermore, overexpressing OsbZIP43 confers plant resistance against insects which can be subverted by miR-7-5P. Our findings suggest a mechanism by which herbivorous insects have evolved salivary miRNAs to suppress plant immunity, expanding our understanding of cross-kingdom RNA interference between interacting organisms.

## Significance

Growing evidence has highlighted the role of small RNAs (sRNA) as trafficking effectors for cross-kingdom RNA interference (RNAi) between interacting organisms. Yet, it remains unknown whether insect-derived miRNAs can serve as the cross-kingdom effectors to regulate plant physiology. Here, we report that *Nilaparvata lugens* secretes sRNAs into host plants to enhance feeding. Specifically, miR-7-5P, which is highly conserved in sequence, serves as a salivary effector targeting multiple host genes. This effector systemically migrates after secretion and may be crucial for insect feeding on rice plants, but not on artificial diets. Our findings illustrate a type of salivary effector that insects may use to manipulate plant immunity.

For millions of years, plants and herbivorous insects have engaged in an intense arms race. To evade or withstand attacks, plants have developed sophisticated immune systems, ranging from signal recognition to defense response activation (1, 2). In turn, insects have evolved multiple effectors to counteract and disrupt plant immunity (3). Saliva, a complex mixture of bioactive components, plays a pivotal role in shaping the intricate interactions between plants and herbivores (4–6). Hitherto, a few proteinic salivary effectors have been well documented. For example, the salivary proteins Bt56 and HARP1 enable insect feeding by manipulating plant hormonal signals (7, 8); the salivary EF-hand-containing proteins from numerous insects suppress plant defenses by binding cytosolic calcium in the host (9, 10). Recently, the discovery of long noncoding RNAs (lncRNAs) in aphid saliva has shed light on their function as virulence factors that enhance aphid fecundity, although their precise mechanism remains unknown (11). Apart from the proteins and lncRNAs mentioned above, our knowledge of other salivary molecules involved in mediating the insect–plant interaction is limited.

MicroRNAs (miRNAs) are a class of small RNAs (sRNAs) widely present in animals, plants, fungi, and viruses (12). Despite their short length, miRNAs/sRNAs exert crucial effects on regulating diverse biological processes in their host organisms, encompassing development, cellular differentiation, and immunity (12, 13). Recent studies have provided mounting evidence supporting that sRNAs also serve as trafficking effectors, enabling the regulation of gene expression across diverse kingdoms through a mechanism known as cross-kingdom RNA interference (RNAi) (14, 15). In the context of fungus–plant interactions, fungal pathogens deliver sRNAs into plant cells to suppress host immune genes and promote infection (16, 17). Conversely, plants release sRNAs into extracellular vesicles to silence virulence genes in the fungal pathogens (18). Similar phenomena have been observed in other cases, including fungus–insect, plant–dodder, and nematode–mammalian interactions, where sRNAs are exchanged to manipulate gene expression and affect the outcome of these interactions (15, 19–22). As for insect–plant interactions, growing evidence has revealed the involvement of plant-derived miRNAs in modulating insect physiology (23, 24). The engineering of plants to express dsRNAs or miRNA mimics targeting insect gene has been regarded as a promising approach of pest management in the agriculture field (25, 26). However, it is still unknown whether insect-derived miRNAs can serve as cross-kingdom effectors to regulate plant physiology. Although such possibility has been proposed, experimental evidence supporting the hypothesis is currently lacking (14, 27).

The brown planthopper, *Nilaparvata lugens*, is a highly damaging pest known to cause substantial yield reductions and economic losses in rice crops. Similar to most phloem sap-sucking insects, *N. lugens* injects a combination of salivary components into the host plant during feeding (28). Although the proteinic components of *N. lugens* saliva and their functions have been extensively studied (29), the presence and the role of other secreted molecules from the insect into host plants remain largely unknown. In this study, the *N. lugens* salivary miRNAs translocated into artificial diets or host plants are investigated. Specifically, a highly abundant and salivary gland–specific miRNA called miR-7-5P was utilized as a molecular probe to explore the mechanism by which this planthopper suppresses plant immunity through cross-kingdom RNAi. miR-7-5P is secreted into host plants during feeding and is shown to target multiple rice genes. Overexpression of miR-7-5P in rice plants benefits insect feeding by suppressing plant immune components, while silencing of miR-7-5P has negative effects on *N. lugens* feeding on rice hosts but not on artificial diets. Our study reveals a type of salivary effector that insects could use to manipulate plant immunity.

## Results

### Identification of Insect miRNAs Translocated into Host Plants.

**Fig. 1.**
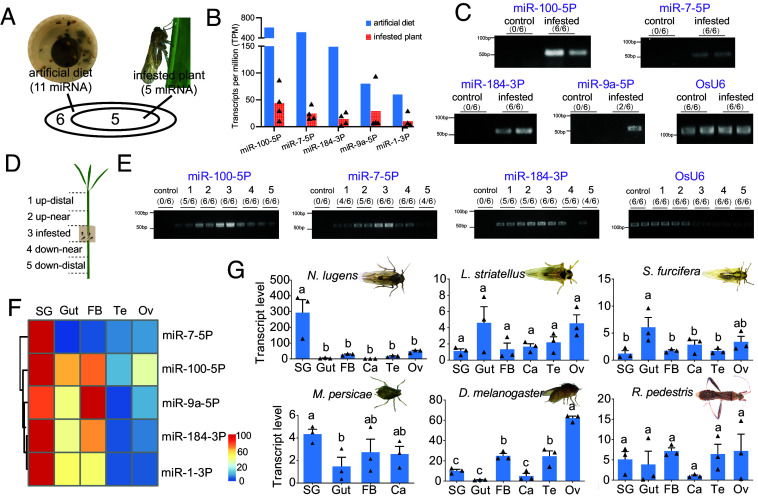
Secretion of *N. lugens* miRNAs into host plants. (*A* and *B*) Identification and relative abundance of miRNAs secreted by *N. lugens* into artificial diets and rice plants. The artificial diets and rice plants infested by *N. lugens* were collected for sRNA sequencing. The noninfested rice plants were used as a control. The abundance of each miRNA was expressed as transcripts per million (TPM). n = 4 independent biological replicates for infested rice plants. (*C*) Validation of *N. lugens* miRNAs secreted into rice plants using stem-loop qRT-PCR. Infested rice plants were analyzed, with untreated rice plants as a control. (*D*) Schematic diagram of five segments in miRNA migration analysis. Insects were confined in the infested segment. (*E*) Detection of miRNAs in the five segments using stem-loop qRT-PCR. The *O. sativa* U6 small nuclear RNA (OsU6) was used to guarantee the RNA quantity. Six independent biological replicates were conducted in (*C*) and (*E*). The presence of target band in each replicate was summarized in the bracket. (*F*) Relative expression levels of miRNAs in different tissues of *N. lugens*. sRNA sequencing was conducted on salivary glands (SGs), guts, fat bodies (Fb), testes (Te), and ovaries (Ov). The expression pattern of each miRNA is illustrated by a heatmap. (*G*) Expression patterns of miR-7-5p homologues in various insect species. Ca, carcasses. *N. lugens*, *Laodelphax striatellus*, *Sogatella furcifera*, *Drosophila melanogaster*, *Riptortus pedestris*, and *Myzus persicae* were selected. Relative expression levels of miRNAs were determined by stem-loop qRT-PCR. Different lowercase letters indicate statistically significant differences at *P* < 0.05 according to the one-way ANOVA test followed by Tukey’s multiple comparisons test. Data are presented as mean ± SEM (n = 3 independent biological replicates).

To explore whether insect miRNAs can be secreted into host plants during feeding, watery saliva was collected from 2,000 *N. lugens* fed on artificial diets for sRNA sequencing. A total of 11 *N. lugens* miRNAs were identified with high abundance (TPM > 10) (*SI Appendix*, Table S1 and Fig. 1 *A* and *B*). The sequences of all 11 miRNAs can be mapped to the *N. lugens* genome but not to the *Oryza sativa* genome, indicating their insect-specific origins (*SI Appendix*, Table S1). Additionally, typical stem-loop structures were identified in these miRNA precursors (*SI Appendix*, Fig. S1). Rice plants infested by *N. lugens* were later subjected to sRNA sequencing. Compared with the noninfested control, five insect miRNAs were detected in infested plants with an average TPM > 10 (*SI Appendix*, Table S2 and Fig. 1 *A* and *B*). The secretion of miRNAs into rice plants was also validated by stem-loop qRT-PCR. The top three abundant miRNAs (miR-100-5P, miR-7-5P, and miR-184-3P) were further confirmed to be secreted in all biological replicates, with target bands being detected in *N. lugens*–infested plants, but not the noninfested controls (Fig. 1*C*). For miR-9a-5P, the target band was detected at a low frequency (2/6, 33.4%), while for miR-1-3P, we failed to detect its presence in infested plants, possibly due to the low secretory quantity. Altogether, three miRNAs were confidently secreted into host plants, including miR-100-5P, miR-7-5P, and miR-184-3P.

To investigate whether insect miRNAs can migrate systemically in plants, rice sheath was divided into five segments, including up-distal segment, up-near segment, infested segment, down-near segment, and down-distal segment (Fig. 1*D*). Insects were confined in the infested segment, and the presence of miR-100-5P, miR-7-5P, and miR-184-3P was detected in five segments, respectively (Fig. 1*E*). The results showed that these three miRNAs can be detected in five segments in most attempts. In a few attempts (1/6 or 2/6), the target bands were not detected in up-distal segment or down-distal segment, possibly due to the low miRNA quantity (Fig. 1*E*). These results suggest that miR-100-5P, miR-7-5P, and miR-184-3P migrate systemically after being secreted into host plants.

The expression patterns of miRNAs in different insect tissues were quantified by sRNA sequencing. Typically, more miRNAs were highly expressed in salivary glands (Fig. 1*F* and *SI Appendix*, Table S3). Among them, miR-7-5P was nearly exclusively expressed in salivary glands. Stem-loop qRT-PCR was further carried out to validate miR-7-5P expression in *N. lugens*, as a result, its transcript was approximately 10-fold higher in salivary glands than other tissues (Fig. 1*G*). miR-7-5P was 24 nt in length and showed a high sequence similarity with other insect miR-7s (*SI Appendix*, Fig. S2). The expression patterns of miR-7 homologues in other insect species were also investigated. The results showed that none of the analyzed insects exhibited salivary gland-specific expression as pronounced as *N. lugens* (Fig. 1*G*). Phylogenetic analysis indicated that the ancestor of *N. lugens* split with *L. striatellus* and *S. furcifera* about 64.4 mya (30), suggesting recent exploration of salivary gland-specific expression of miR-7-5P in *N. lugens*.

### miR-7-5P Is Critical for Insect Feeding on Rice Plants but Not on Artificial Diets.

**Fig. 2.**
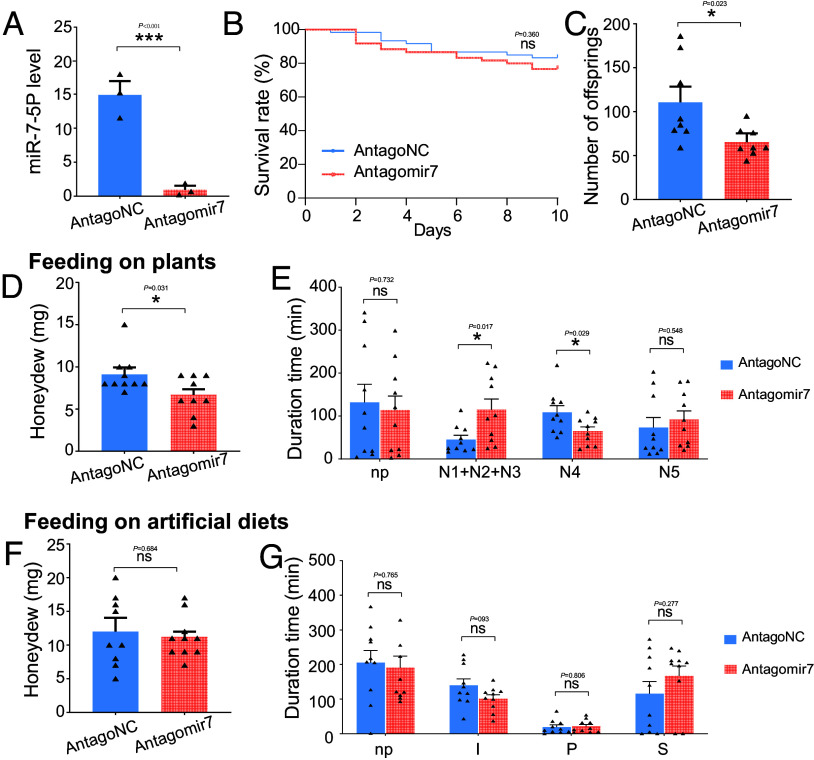
Effects of miR-7-5P suppression on *N. lugens* performance. (*A*) Successful suppression of miR-7-5P expression using antagomir7. AntagoNC served as the negative control. n = 3 independent biological replicates. (*B*–*E*) Effects of miR-7-5P suppression on insects feeding on rice variety cv. Nipponbare. The parameters assessed included insect survivorship (*B*), reproduction (*C*), honeydew excretion (*D*), and feeding behavior (*E*). Insect feeding behavior on rice plants can be classified into nonpenetration (np), pathway duration (N1+N2+N3), phloem sap ingestion (N4), and xylem sap ingestion (N5) phases. For survival analysis, n = 60 individuals in each treatment. For reproduction, honeydew and EPG analyses, n = 8, n = 9, and n = 10 independent biological replicates, respectively. (*F* and *G*) Effects of miR-7-5P suppression on insect honeydew excretion (*F*) and feeding behavior (*G*) when feeding on artificial diets. Insect feeding behavior on artificial diets can be classified into np, penetration initiation (p), salivation and stylets movement (S), and ingestion (I) phases. For honeydew analysis in (*F*), n = 9 and n = 10 independent biological replicates for antagoNC and antagomir7 treatment, respectively. For EPG analysis in (*G*), n = 10 independent biological replicates. Survivorship differences were assessed using the log-rank test. The EPG data were first checked for normality and homogeneity of variance, and data not fitting a normal distribution were subjected to log10 transformation. Differences in gene expression, reproduction, honeydew excretion, and EPG recording were determined by two-tailed unpaired Student’s *t* test. ***P < 0.001; *P < 0.05; ns, not significant. Data in (*A* and *C*–*G*) are presented as mean ± SEM.

To investigate whether miR-7-5P affected insect feeding on rice hosts, *N. lugens* were treated with antagomir7 (an miR-7-5P inhibitor whose sequence is complementary to the mature miR-7-5P) or antagoNC (a negative control), and reared on the rice variety cv. Nipponbare and cv. Xiushui, respectively. Silencing efficiency was determined on the fourth day postinjection, and the results showed that antagomir7 significantly suppressed the expression of miR-7-5P by more than 90% compared with the antagoNC control (Fig. 2*A*). For the treated insects feeding on Nipponbare plants, silencing miR-7-5P did not influence the insect survivorship, with nearly all tested insects surviving 10 d posttreatment (Fig. 2*B*). However, the antagomir7-treated insects produced less offspring (decreased by 41%) and excreted less honeydew (decreased by 27%) than those of the control (Fig. 2 *C* and *D*). Honeydew is an indicator of feeding status in planthopper species (31). The decreased honeydew excretion indicated the impaired feeding process. Therefore, electrical penetration graph (EPG) was further employed to monitor the *N. lugens* feeding behavior (*SI Appendix*, Fig. S3*A*). The results showed that antagomir7-treated insects exhibited a significant decrease (by 40%) in phloem sap ingestion and a significant increase (by 153%) in pathway duration (Fig. 2*E*). Similar results were observed for the treated *N. lugens* feeding on Xiushui plants (*SI Appendix*, Fig. S4), indicating a potential role of miR-7-5P in insect feeding on plants.

The effects of miR-7-5P on artificial diets were also investigated. We did not observe significant difference in honeydew excretion between antagomir7- and antagoNC-treated *N. lugens* (Fig. 2*F*). In EPG analysis, the insect feeding behavior can be classified into nonpenetration (np), penetration initiation (p), salivation and stylets movement (S), and ingestion (I) phases (32) (*SI Appendix*, Fig. S3*B*). There was no significant difference in either phase between two treatments (Fig. 2*G*), indicating that miR-7-5P was unnecessary in insect feeding on artificial diets.

### miR-7-5P Targets Plant Genes.

We utilized three miRNA target prediction software applications to identify potential targets of miR-7-5P. The results revealed that miR-7-5P exhibited complementary base pairing with 52 rice genes (*SI Appendix*, Table S4). Of them, bZIP transcription factor 43 (bZIP43, Os02g49560), NBS-LRR-like resistance protein (Os03g38330), disease resistance protein RPM1 (Os04g02860), BTB/POZ domain containing protein (Os10g30040), lipoxygenase (Os12g37260), and receptor kinase 2 (Os10g18990), which have been widely reported to be involved in plant immunity (33–36), were selected for interaction validation. We cloned DNA fragments surrounding the predicted target sites and ligated them to the 3′ end of the GFP gene. Vectors encoding GFP protein alone or recombinant β-glucuronidase (GUS)-GFP protein were served as negative controls. The recombinant plasmids were transformed into the *Agrobacterium tumefaciens*, and infiltrated into *Nicotiana benthamiana* leaves with *A. tumefaciens* containing p35S: miR-7-5P at different concentrations. miR-7-5P level in *N. benthamiana* leaves was changed as expected (*SI Appendix*, Fig. S5). Fluorescence analysis demonstrated that all the six tested genes displayed weaker GFP fluorescence upon coexpression with miR-7-5P than the controls (*SI Appendix*, Fig. S5). Western blotting assay further confirmed the decrease of target bands with the presence of miR-7-5P (*SI Appendix*, Fig. S5). In contrast, miR-7-5P did not influence the GFP fluorescence or protein amount of the controls that did not contain target sequences (*SI Appendix*, Fig. S5).

The bZIP gene family plays a crucial role in stress responses (33, 37). psRNAtarget software revealed the highest reliability for the pairing of miR-7-5P with OsbZIP43 (*SI Appendix*, Table S4). First, we investigate the role of OsbZIP43 on rice defense against *N. lugens*. The transgenic rice plant overexpressing OsbZIP43 was constructed, and two independent homozygous lines (*oebZIP*#1 and *oebZIP*#2) were selected (*SI Appendix*, Fig. S6 *A* and *B*). The empty vector (EV) transgenic plant was used as a negative control. Bioassay results showed that *N. lugens* excreted less honeydew and produced less offspring on *oebZIP* plants than on control plants (*SI Appendix*, Fig. S6 *C* and *D*), suggesting that OsZIP43 contributes to plant defense against *N. lugens*. Therefore, we select OsbZIP43 in subsequent experiments to investigate the potential role of miR-7-5P in insect–plant interactions.

**Fig. 3.**
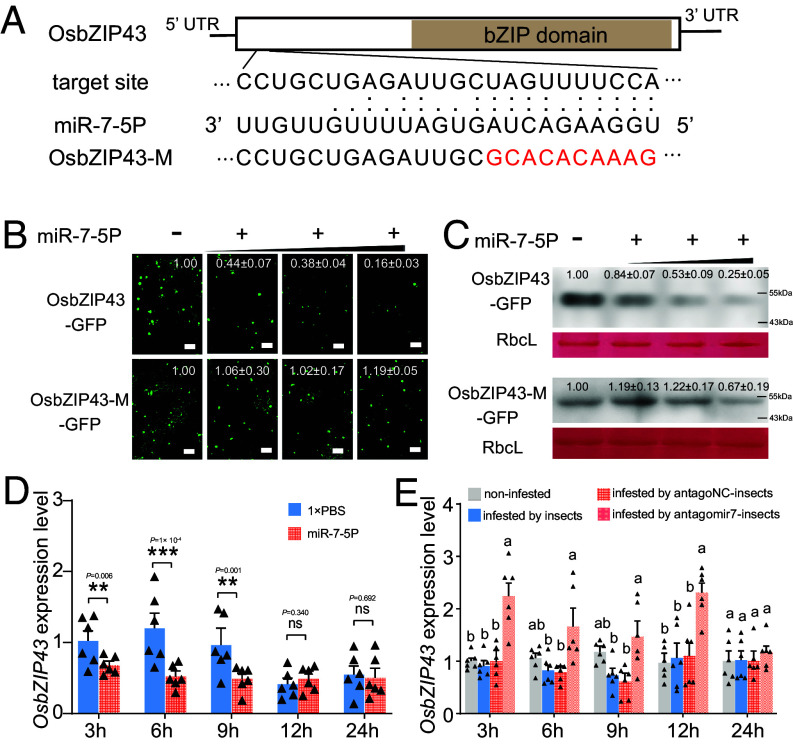
miR-7-5P suppresses the expression of OsbZIP43, but not the OsbZIP43-M. (*A*) A schematic diagram showing the base pairing between miR-7-5P and OsbZIP43. The OsbZIP43 sequence with a substituted base-pairing region (OsbZIP43-M) was constructed. The substituted bases are labeled in red. (*B* and *C*) miR-7-5P significantly reduced the green fluorescence and protein level of OsbZIP43-GFP, but not those of OsbZIP43-M-GFP. At 48 h after coinfiltration, green fluorescence from OsbZIP43-GFP or OsbZIP43-M-GFP was observed in *N. benthamiana* leaves (*B*), and the protein levels of GFP-fused proteins were determined by western blotting assay (*C*). Rubisco staining (RbcL) was conducted to visualize the sample loading amount. (Scale bar, 100 μm.) Three independent biological replicates were performed, and the representative fluorescence and western blotting images are displayed. Fluorescence intensity in (*B*) and band density in (*C*) were measured using ImageJ. The intensity/density values from three biological replicates were calculated and the mean value in the controls was set at 1.0. The small triangle indicates the different concentrations (OD_600_ = 0.05, 0.3, and 1.0) of *Agrobacterium* harboring p35S: miR-7-5P. (*D*) Relative transcript level of *OsbZIP43* after injection of miR-7-5P into rice plants. The miR-7-5P or 1× PBS was injected into rice plants using a capillary, and the expression of *OsbZIP43* was determined by qRT-PCR. (*E*) Relative transcript level of *OsbZIP43* after *N. lugens* infestation. The rice plants were infested by untreated, antagoNC-treated, and antagomir7-treated *N. lugens*, and the expression of *OsbZIP43* was determined by qRT-PCR. The untreated rice plant was used as a control. Data in (*D*) and (*E*) are presented as mean ± SEM (n = 6 independent biological replicates). *P*-value in (*D*) was determined by two-tailed unpaired Student’s *t* test. Different lowercase letters in (*E*) indicate statistically significant differences at *P* < 0.05 according to the one-way ANOVA test followed by Tukey’s multiple comparisons test.

The target sites of miR-7-5P are located at the 5′ terminal of full open reading frame (ORF) of OsbZIP43, out of the bZIP domain (Fig. 3*A*). First, we ligated the full ORF of OsbZIP43 (Os02g49560) to GFP, and coexpressed with p35S: miR-184-3P (negative control) or p35S: miR-7-5P in *N. benthamiana*. GFP fluorescence was mainly observed in the nucleus of leaves coexpressing OsbZIP43-GFP and different concentrations of miR-184-3P control (*SI Appendix*, Fig. S7*A*). By contrast, a dose-dependent decrease in OsbZIP43-GFP fluorescence was observed after coexpression with miR-7-5P at different concentrations (*SI Appendix*, Fig. S7*A*). Western blotting results showed that OsbZIP43-GFP was negatively regulated by miR-7-5P, but not by miR-184-3P (*SI Appendix*, Fig. S7*B*). Then, the target site of OsbZIP43 was mutated (OsbZIP43-M) (Fig. 3*A*), and coexpressed with p35S: miR-7-5P. The result showed that miR-7-5P failed to influence the fluorescence of OsbZIP43-M-GFP (Fig. 3*B*). Additionally, the transcript and protein levels of OsbZIP43-M-GFP were not significantly affected by miR-7-5P at a low concentration (Fig. 3*C* and *SI Appendix*, Fig. S8). At a high concentration of miR-7-5P, reduced protein level of OsbZIP43-M-GFP was still observed (Fig. 3*C*), which might be caused by the changed plant physiology or incomplete target mutation.

In most cross-kingdom RNAi studies, miRNAs from the donor usually suppress target genes in the recipient. In this study, miR-7-5P or 1× PBS was injected into rice plants using a capillary at first, and then the transcript level of *OsbZIP43* was detected at 3, 6, 9, 12, and 24 h postinjection. The results showed that miR-7-5P significantly suppressed *OsbZIP43* expression at 3, 6, and 9 h postinjection (Fig. 3*D*), suggesting that miR-7-5P potentially plays a role on *OsbZIP43* expression. Afterward, the transcript level of *OsbZIP43* in response to *N. lugens* infestation was determined. However, there was no statistically significant change in the transcript level of *OsbZIP43* when comparing *N. lugens*–infested rice with those in noninfested rice (Fig. 3*E*), although the *OsbZIP43* level was lower in infested plants than the controls at 6 and 9 h after *N. lugens* infestation (Fig. 3*E*). Given that bZIP genes are widely reported to be induced upon pathogen infection (33), we compared the rice plants infested by antagoNC- and antagomir7-treated *N. lugens*. The results showed that antagomir7-treated insects significantly induced the expression of *OsbZIP43* at 3, 6, 9, and 12 h postinfestation, while antagoNC-treated counterparts did not significantly affect *OsbZIP43* expression (Fig. 3*E*). These results suggest that OsbZIP43 is potentially induced upon *N. lugens* in deficient of miR-7-5P secretion, while the presence of miR-7-5P may rescue this effect.

### Overexpression of miR-7-5P Attenuates Plant Defenses.

**Fig. 4.**
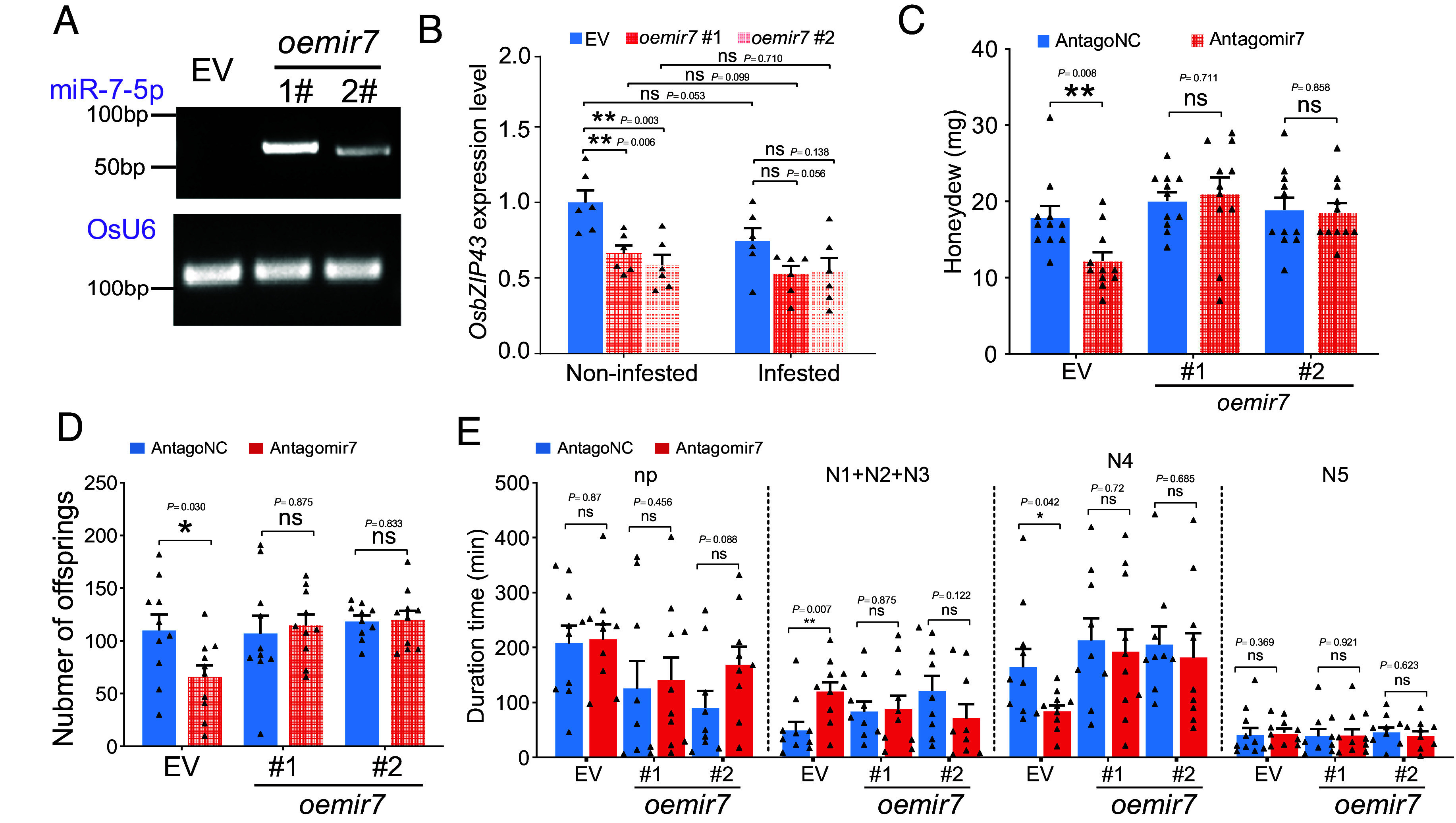
Effects of miR-7-5P overexpression on rice plants. (*A*) Detection of miR-7-5P in transgenic plant *oemir7*#1 and *oemir7*#2, with EV transgenic plants being used as a control. The *O. sativa* U6 small nuclear RNA (OsU6) was used to guarantee the RNA quantity. Experiments were repeated three times with the similar results. (*B*) Comparison of *OsbZIP43* expression levels between *oemir7* and EV plants using qRT-PCR (n = 6 independent biological replicates). The infested samples were collected at 6 h post *N. lugens* infestation. (*C*–*E*) Effects of miR-7-5P suppression on insect honeydew excretion (n = 11 independent biological replicates), reproduction (n = 10 independent biological replicates), and feeding behavior (n = 10 independent biological replicates) when feeding on *oemir7* and EV plants. The EPG data in (*E*) were first checked for normality and homogeneity of variance, and data not fitting a normal distribution were subjected to log10 transformation. *P*-values were determined by two-tailed unpaired Student’s *t* test. ***P < 0.001; **P < 0.01; *P < 0.05; ns, not significant. Data are presented as mean ± SEM.

To investigate the role of miR-7-5P in host plants, we constructed the transgenic rice plant overexpressing miR-7-5P. Two independent homozygous lines (*oemir7*#1 and *oemir7*#2) were selected (Fig. 4*A*). Compared with EV plants, *oemir7*#1 and *oemir7*#2 plants untreated or infested by *N. lugens* showed the decreased expression of *OsbZIP43* (Fig. 4*B*). In EV and *oemir7*#1 plants, *N. lugens* infestation decrease the transcript level of *OsbZIP43*, although no statistical significance was detected (Fig. 4*B*). Bioassay results showed that antagomir7-treated *N. lugens* excreted less honeydew and produced less offspring on EV plants (Fig. 4 *C* and *D*), consistent with the results on WT plants (Fig. 2 and *SI Appendix*, Fig. S4). For antagomir7- and antagoNC-treated *N. lugens* feeding on *oemir7* plants, we did not observe a significant difference in honeydew excretion or reproduction (Fig. 4 *C* and *D*). The impaired feeding of antagomir7-treated *N. lugens* on EV plants was validated by EPG analysis, while there was no significant difference between antagomir7- and antagoNC-treated *N. lugens* feeding on *oemir7* plants (Fig. 4*E*). Noteworthily, *N. lugens* excreted significantly increased honeydew after feeding on *oemir7* plants relative to on EV plants (*SI Appendix*, Fig. S9*A*). For reproduction analysis, *N. lugens* had more offspring on *oemir7* plants, although the difference was not statistically significant (*SI Appendix*, Fig. S9*B*). These results suggest that *oemir7* plants are beneficial for insects and can rescue the impaired feeding performance of miR-7-5P-silenced insects.

**Fig. 5.**
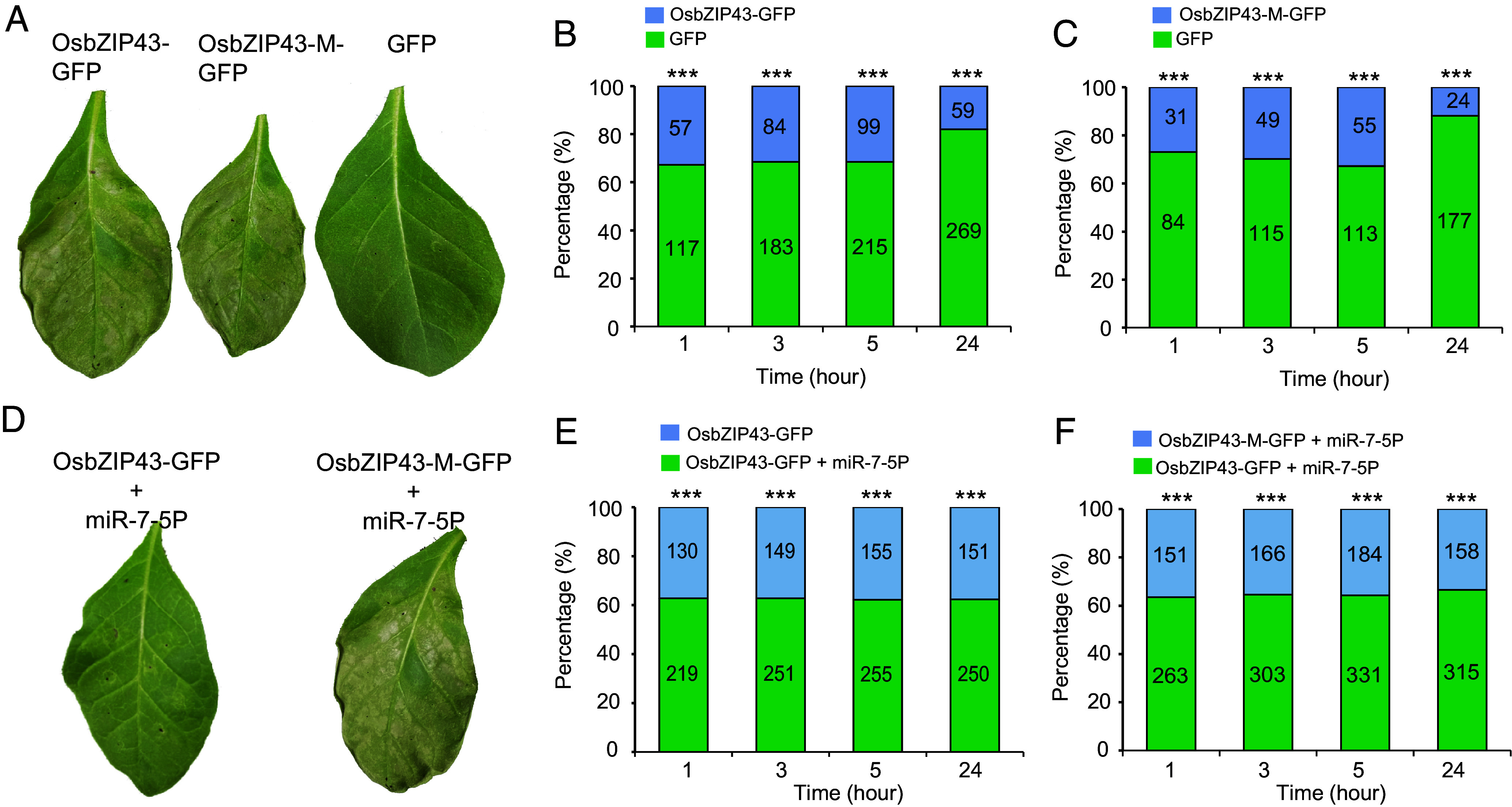
Effects of OsbZIP43 and miR-7-5P on plant defenses. (*A*) Induction of leaf necrosis by the overexpression of OsbZIP43-GFP and OsbZIP43-M-GFP in *N. tabacum*. Agrobacteria carrying OsbZIP43-GFP, OsbZIP43-M-GFP, and GFP were infiltrated into *N. tabacum* leaves, and the phenotype was photographed at 4 d postinfiltration. (*B*) Host selection analysis between *N. tabacum* leaves expressing GFP and OsbZIP43-GFP. (*C*) Host selection analysis between *N. tabacum* leaves expressing GFP and OsbZIP43-M-GFP. (*D*) miR-7-5P rescued the necrosis phenotype caused by OsbZIP43-GFP overexpression, but not by OsbZIP43-M-GFP overexpression. (*E*) Host selection analysis between *N. tabacum* leaves expressing OsbZIP43-GFP and OsbZIP43-GFP/ miR-7-5P. (*F*) Host selection analysis between *N. tabacum* leaves expressing OsbZIP43-M-GFP/ miR-7-5P and OsbZIP43-GFP/ miR-7-5P. There was no significant necrosis effect on leaves at 2 d postinfiltration, and these leaves were used for host selection analysis. The number of whiteflies on each leaf is illustrated in a bar graph. *P*-values were determined by the Chi-square test. ***P < 0.001. At least 10 independent biological replicates were performed in (*A*) and (*D*), and the representative images are displayed.

Subsequently, we used the tobacco-whitefly system to investigate the effect of miR-7-5P on OsbZIP43-mediated plant defenses. The OsbZIP43-GFP, OsbZIP43-M-GFP, and GFP were overexpressed in *Nicotiana tabacum*, respectively. Necrosis, a by-product of plant immunity, was observed in OsbZIP43-GFP- and OsbZIP43-M-GFP-expressing leaves approximately 4 d postinfiltration, but not in the GFP-expressing one (Fig. 5*A*). At 48 h postinfiltration, no necrosis effect was detected in OsbZIP43-GFP- or OsbZIP43-M-GFP-expressing leaves, with no detectable cell morphological change in the infiltrated leaves (*SI Appendix*, Fig. S10). In contrast, leaves infiltrated with InF1, a necrosis-inducing protein from *Phytophthora infestans* (38), exhibited significant cell morphological changes (*SI Appendix*, Fig. S10). Therefore, a host selection experiment was conducted at 48 h postinfiltration. The results demonstrated that whiteflies preferred to settle on GFP-expressing leaves, but not on OsbZIP43-GFP- or OsbZIP43-M-GFP-expressing ones (Fig. 5 *B* and *C*), indicating the enhanced plant defense responses in OsbZIP43- and OsbZIP43-M-expressing plants. Subsequently, p35S: miR-7-5P was coexpressed with OsbZIP43-GFP or OsbZIP43-M-GFP in *N. tabacum* leaves (*SI Appendix*, Fig. S11). miR-7-5P significantly rescued the necrosis effect induced by OsbZIP43-GFP overexpression, but not OsbZIP43-M-GFP overexpression (Fig. 5*D*). Additionally, whiteflies preferred to settle on tobacco leaves coexpressing OsbZIP43-GFP and miR-7-5P compared with those coexpressing OsbZIP43-M-GFP and miR-7-5P, or expressing OsbZIP43-GFP alone (Fig. 5 *E* and *F*). These results suggested that miR-7-5P may be capable of attenuating OsbZIP43-trigered plant defenses.

## Discussion

Since the initial studies on cross-kingdom RNAi in fungus–plant interactions, this regulatory mechanism has attracted increasing attention and has been revealed in various interaction systems (14). However, to our knowledge, the involvement of insect-derived miRNAs in modulating plant physiology is poorly understood. Here, we reported that *N. lugens* potentially employed salivary miRNAs to silence a bZIP43 gene involved in plant defense, revealing a cross-kingdom defense strategy.

While *N. lugens* encodes hundreds of miRNAs, only a minority of these miRNAs are secreted into saliva. Among the five salivary miRNAs abundantly detected in both watery saliva and infested plants, four exhibit the highest expression levels in salivary glands (Fig. 1*F*). As an important secretory tissue, the salivary gland serves as the primary source of salivary components (39). In *N. lugens*, numerous salivary proteins were abundantly and specifically expressed in salivary glands (28). Our findings highlighted the resemblance in expression patterns between miR-7-5P and salivary proteins, indicating the potential role of miR-7-5P in insect feeding. Notably, although the sequence of miR-7-5P was conserved across different insects (*SI Appendix*, Fig. S2), its expression patterns varied significantly (Fig. 1*G*). For example, in *D. melanogaster*, miR-7-5P was primarily associated with insect development, such as insulin regulation and wing growth (40, 41), and it was expressed at a low level in salivary glands (Fig. 1*G*). In the phylogenetically close relatives *L. striatellus* and *S. furcifera*, miR-7-5P did not exhibit salivary gland-specific expression (Fig. 1*G*). Typically, *N. lugens* is a monophagous pest that exclusively feeds on rice plants, whereas *L. striatellus* and *S. furcifera* are oligophagous and capable of feeding on a variety of grass plants (42). This suggests that *N. lugens* may have evolved the salivary gland-specific expression of miR-7-5P through long-term coevolution with its rice hosts.

Cross-kingdom miRNAs regulate target gene expression at both the transcriptional and posttranscriptional levels in diverse biological interactions (14, 15). The importance of miR-7-5P in *N. lugens* feeding on rice hosts, but not on the artificial diet, prompted us to study its role in regulating plant immunity (Fig. 2). The bZIP gene family is well known to be essential for plants to overcome biotic stresses, with transcripts significantly induced upon infection by various pathogens (33, 43). Our study demonstrated that rice plants potentially up-regulate the expression of OsbZIP43 in response to insect infestation, but this induction effect was counteracted by the presence of salivary miR-7-5P (Fig. 3 *D* and *E*). Consistent with defensive roles of bZIP genes, overexpression of OsbZIP43 in plants negatively affected insect feeding (*SI Appendix*, Fig. S6 and Fig. 5). The target sites of miR-7-5P were located outside the bZIP domain of OsbZIP43. Experimental mutations of these sites did not affect the immune-triggering properties of this gene, but miR-7-5P failed to inhibit the plant defense mediated by OsbZIP43-M (Fig. 5), highlighting the importance of this pair-wise region in this interaction. Future studies are required to demonstrate that insect feeding actually increases OsbZIP43 expression and that direct abrogation of this transcription factor decreases a plant’s resistance to insect attack.

In addition to OsbZIP43, miR-7-5P also silenced host genes associated with signal recognition and defense activation (*SI Appendix*, Table S4). Herbivorous hemipteran insects probe host plants with their piercing-sucking mouthparts, causing inevitable damage to plant cells (39). Host plants can recognize components present in the insect saliva, leading to the activation of pattern recognition receptors (PRRs)-triggered immunity (PTI) and nucleotide-binding domain, leucine-rich repeat domain-containing receptors (NLRs)-mediated effector-triggered immunity (ETI) (44, 45). Pathogenic effectors, particularly those of protein origin, have been widely reported to target plant PRRs or NLRs. For example, the bacterial effector protein XopK possesses the E3 ubiquitin-ligase activity, which degrades rice receptor kinase OsSERK2 (46). Similarly, the fungal effector protein NIS1 inhibits the activity of PRR-associated kinases BAK1 and BIK1 (47). Our study demonstrated that miR-7-5P targeted multiple host genes, including NBS-LRR-like resistance protein Os03g38330 and receptor kinase Os10g18990, suggesting that different invaders independently evolved effectors to counteract plant immunity.

Collectively, findings in this study confirm that salivary miRNAs are secreted into host plants during insect feeding. Among these miRNAs, the sequence-conserved miR-7-5P appears to be a newly evolved salivary effector that targets multiple host genes to suppress the plant immunity (*SI Appendix*, Fig. S12). This study highlights the significance of cross-kingdom RNAi in insects adapting to plant hosts.

## Materials and Methods

### Insects and Plants.

The *N. lugens* strain used in this study was originally collected from a rice field at Huajiachi Campus, Zhejiang University, Hangzhou, China. The insects and rice plants were maintained in a climate chamber at 25 ± 1 °C, with 70 to 80% relative humidity, and a light/dark photoperiod of 16/8 h. In addition, *N. benthamiana* and *N. tabacum* K326 plants were kept at 23 ± 1 °C under a light/dark photoperiod of 16/8 h.

### Collection of Saliva in Artificial Diets.

The fifth instar *N. lugens* nymphs were collected and subjected to a 24 h starvation period with only water provided. Subsequently, the insects were transferred to sterile diets containing 2.5% sucrose, which was added between two layers of Parafilm Laboratory Film (Bemis NA, Neenah, WI). Following a 24 h feeding period, the sucrose solution was collected using a needle and mixed with TRIzol Reagent (#10296018, Invitrogen, Carlsbad, CA) at a ratio of approximate 1:1. A total of 2,000 nymphs were utilized for saliva collection.

### Collection of *N. lugens*–Infested Plants.

Insects inject saliva into host plants during feeding. To identify salivary components in infested plants cv. Nipponbare, approximately 50 fifth instar *N. lugens* nymphs were confined within a 3 cm rice stem. Following a 24 h feeding period, the outermost leaf sheath in the infested region was cut and grinded with liquid nitrogen. Subsequently, the samples were homogenized in TRIzol Reagent for RNA extraction. Rice plants without *N. lugens* infestation were used as controls. Four independent biological replicates were performed.

### Migration of miRNAs in Plants.

To investigate whether insect miRNAs could migrate from infested site to noninfested site, the rice sheath was divided into five segments (Fig. 1*D*). Approximately 50 fifth instar *N. lugens* nymphs were confined in the infested segment and allowed feeding for 24 h. Subsequently, rice sheath was cut into five segments and homogenized in TRIzol Reagent as described above. Six independent biological replicates were performed.

### Small RNA (sRNA) Sequencing and Analysis.

Different tissue samples from *N. lugens* fat bodies (20), guts (30), and salivary glands (40) were dissected from the fifth instar nymphs in a phosphate-buffered saline (PBS) solution (137 mM NaCl, 2.68 mM KCl, 8.1 mM Na_2_HPO_4_, and 1.47 mM KH_2_PO_4_ at pH7.4) using a pair of forceps (Ideal-Tek, Switzerland). Similarly, testes (20) and ovaries (10) were collected from adult male and female *N. lugens*, respectively. The number of insects in each sample was given in the parentheses above. Total RNA was extracted following the recommended protocols of the manufacturer. Once the integrity and quantity of RNA samples were determined, they were sent to Novogene Company in Tianjin, China, for sRNA sequencing. Briefly, the sRNA libraries were prepared using the Illumina TruSeq Small RNA Sample Preparation Kit (#RS-200, Illumina, San Diego, CA). High-throughput sequencing was conducted on the Illumina HiSeq 2500 platform. The resulting raw data underwent quality trimming, which involved the removal of adapters and low-quality sequences, using the Cutadapt tool (48). Furthermore, *N. lugens* miRNAs were identified based on a previously described method (49). The mapped miRNAs were then subjected to BLAST analysis against the *N. lugens* (https://www.ncbi.nlm.nih.gov/datasets/genome/GCF_014356525.2/) and the *O. sativa* (https://data.jgi.doe.gov/search?q=Oryza+sativa&expanded=Phytozome-323) genomes. Subsequently, miRNA frequencies were calculated using a Perl script (50). The relative level of each miRNA was presented as transcripts per million (TPM). miRNAs with TPM > 10 were considered high-abundance miRNAs. The secondary structures of miRNA precursors were predicated using sRNAminer software (https://github.com/kli28/sRNAminer). The sequencing data were submitted to the National Genomics Data Center under accession number PRJCA018311.

### Tissue Collection from Different Insects.

Insect tissues were collected following the similar procedure as described above. For *N. lugens*, *L. striatellus*, *S. furcifera*, and *D. melanogaster*, the number of insects used for salivary gland, gut, fat body, carcass, testis, and ovary collection was approximately 40, 30, 20, 20, 20, and 10, respectively. For *Myzus persicae*, approximately 60 salivary glands, 50 guts, 20 fat bodies, and 20 carcasses were used for sample collection. For *Riptortus pedestris*, approximately 10 salivary glands, 5 guts, 5 fat bodies, 5 carcasses, 5 testes, and 5 ovaries were used for sample collection. The isolated tissues were transferred to TRIzol Reagent immediately and homogenized before RNA extraction.

### qPCR Analysis of miRNAs and mRNAs.

Stem-loop qRT-PCR was utilized to evaluate the relative expression levels of miR-7-5P in rice and various insect tissues. Initially, total miRNAs were extracted using the EasyPure miRNA Kit (#R10215, TransGen, Beijing, China) according to the manufacturer’s protocol. Subsequently, 0.5 μg miRNAs were used to synthesize the first-strand cDNA using the miRNA first Strand cDNA Synthesis Kit (#MQ101, Vazyme, Nanjing, China). Following cDNA synthesis, stem-loop qRT-PCR was performed on the Applied Biosystems platform using the Hieff miRNA Universal qPCR SYBR Master Mix (#11170ES03, Yeasen, Shanghai, China).

To analyze the mRNA level, total RNA was extracted as described above. The first-strand cDNA was reverse-transcribed from total RNA using HiScript II Q RT SuperMix (#R212-01, Vazyme). Subsequently, qRT-PCR was run on a Roche Light Cycler® 480 Real-Time PCR System (Roche Diagnostics, Mannheim, Germany) using the SYBR Green Supermix Kit (#11202ES08, Yeasen).

The primers used in qRT-PCR assay are listed in *SI Appendix*, Table S5. The U6 small nuclear RNA from *N. lugens* and *O. sativa* served as internal controls for miRNA analysis, while the actin genes from *N. lugens*, *O. sativa*, and *N. benthamiana* were used as internal controls for mRNA analysis. A relative quantitative method (2^−ΔΔCt^) was used to calculate the quantitative variation (51). Three or six independent biological replicates with each repeated twice were performed.

### Silencing of miRNA in *N. lugens*.

The miR-7-5P inhibitor, antagomir7 (50 ng/μL, 5′-AACAACAAAAUCACUAGUCUUCCA-3′, all nucleic acids undergo methylation modification, two and four bases at the 5′ and 3′ ends undergo thio-modification, respectively, RiboBio Company, Guangzhou, China), was employed to silence miR-7-5P, while the antagoNC (50 ng/μL, micrOFF antagomir NC 24, #miR3N0000002-4-5, RiboBio) served as a negative control. Before injection, the insects were anesthetized with carbon dioxide for 5 to 10 s. Afterward, the synthetic RNA (approximately 0.05 µL per insect) was injected into the mesothorax of each insect using a FemtoJet (Eppendorf-Netheler-Hinz, Hamburg, Germany). Subsequently, the treated insects were placed on 4- to 5-leaf stage rice seedlings for 24 h, and only living insects were selected for further investigation. The silencing efficiency was determined on the fourth day postinjection using the stem-loop qRT-PCR method as described above. For survival, honeydew, and EPG analyses, third instar nymphs were used. For reproductive analysis, newly emerged female adults were utilized.

### Injection of miRNA into Rice Plants.

miR-7-5P was synthesized by RiboBio Company and dissolved in a 1× PBS solution. Prior to injection, the capillary (VitaiSense Scientific, Wuhan, China) was pulled to generate microinjection needles using a P-97 Micropipette Puller (Sutter Instrument, Novata). Then, miR-7-5P (50 ng/μL) was loaded into a capillary and injected into the outermost leaf sheath of rice plants using the FemtoJet (Eppendorf-Netheler-Hinz). One plant was pierced for five times and injected with approximately 0.2 µL solution for each. The 1× PBS solution was used as a negative control. The treated leaf sheaths were collected at 3, 6, 9, 12, and 24 h postinjection. Six independent biological replicates were performed.

### Insect Bioassays.

In the survival analysis, a group of 20 treated insects were placed on 4- to 5-leaf stage rice seedlings in a climate chamber. The mortality rate for each treatment was recorded for 10 consecutive days. Three independent replications were performed.

For the honeydew analysis, 10 treated insects were enclosed within a parafilm sachet (Bemis NA) attached to the host plant stems. The insects were allowed feeding for 24 h, and the excreted honeydew was measured by weighing the parafilm sachet before and after feeding using an electronic balance with an accuracy of 0.001 g (Sartorius, Beijing, China). Nine to twelve biological replicates were performed for each treatment.

In the reproductive analysis, one treated female insect was paired with one untreated male. The insect pair was later transferred to a plastic cup containing 10 rice seedlings and was allowed to oviposit for 10 d. The number of hatched offspring was counted every day until no offspring emerged for a consecutive period of 3 d. One plastic cup containing a pair of male and female insects was regarded as a replicate, and 8 to 15 replicates were conducted for each treatment. The sterile females were excluded from data analysis.

### EPG Recording Analysis.

The direct-current EPG amplifier (Model Giga-8d) (Wageningen Agricultural University, Wageningen, Netherlands) was utilized to monitor the insect feeding behaviors. To prepare for the monitoring, the fifth instar nymphs were anesthetized with CO_2_ for 10 s. A gold wire (Wageningen Agricultural University, diameter: 20 mm, length: 5 cm) was used to connect the insect abdomen to the EPG amplifier using a water-soluble silver conductive glue (Wageningen Agricultural University). To analyze the insect feeding on rice plants, a copper wire (diameter: 2 mm, length: 10 cm) was inserted into the soil with a rice plant. To analyze the insect feeding on artificial diets, the copper wire was inserted into the Parafilm-covered petri dish that contained 2.5% sucrose solution. The EPG recording system was then performed for 8 h within a Faraday cage (120 cm × 75 cm × 67 cm, Dianjiang, Shanghai, China). The gain of the amplifier was set at 50×, and the output voltage was adjusted between −5 V and +5 V to optimize the recording conditions.

The output data were analyzed with PROBE 3.4 software (Wageningen Agricultural University). As for *N. lugens* feeding on rice plants, the EPG waves were classified into nonpenetration (np), pathway duration (N1+N2+N3), phloem sap ingestion (N4), and xylem sap ingestion (N5) as previously described (28). In the case of *N. lugens* feeding on artificial diets, the EPG waves were classified into nonpenetration (np), penetration initiation (p), salivation and stylets movement (S), and ingestion (I) phases (32).

### Prediction of miRNA Targets.

To predict the genes targeted by miR-7-5P, three computational target prediction algorithms were adopted, including the psRNATarget (https://www.zhaolab.org/psRNATarget/analysis, accessed on February 2022, default parameters, expectation threshold < 5.0, allowed two mismatches), miRanda (http://www.bioinformatics.com.cn/local_miranda_miRNA_target_prediction_120, accessed on February 2022, default parameters, score threshold > 140, energy threshold < 15 kcal/mol), and RNAhybrid (https://bibiserv.cebitec.uni-bielefeld.de, accessed on February 2022, default parameters, energy threshold < 15 kcal/mol). Only the target genes commonly predicted by these three algorithms were selected for further analyses.

### Transient Expression in *N. benthamiana* and *N. tabacum* Leaves.

To investigate the role of miRNAs in regulating the target gene expression, we inserted the looped miR-7-5P (p35S: miR-7-5P) or miR-184-3P (p35S: miR-184-3P) sequences downstream of a CaMV 35S promoter at the BsaI/EcoRI site in the pBWA(V)HS vector. Similarly, we cloned the target genes or DNA fragments surrounding the predicted target sites into the 3′ end of the GFP gene in the Bin-GFP vector to generate recombinant GFP: target plasmids. Each of these expression vectors was then individually transformed into the *A. tumefaciens* GV3101 by the heat transfer method. Thereafter, *Agrobacterium* harboring GFP: target was suspended in the infiltration buffer (10 mM MgCl_2_, 0.5 mM MES, and 0.2 mM acetylacetone) to OD_600_ = 1.0 and mixed with different concentration of *Agrobacterium* harboring p35S: miR-7-5P or p35S: miR-184-3P. In *SI Appendix*, Fig. S5, the concentrations were set at OD_600_ = 0, 0.3, and 1.0; while in Fig. 3 and *SI Appendix*, Fig. S7, the concentrations were set at OD_600_ = 0, 0.05, 0.3, and 1.0. Subsequently, the *Agrobacterium* mixture was infiltrated into the leaves of *N. benthamiana*. After a 48 h incubation period, the infiltrated leaves were harvested, and the fluorescence signal derived from GFP expression was observed using a Leica confocal laser-scanning microscope SP8 (Leica Microsystems). To compare the fluorescence intensity among different treatments, the fluorescence was captured under the same parameters (intensity = 1.0; zoom in = 1.6). The fluorescent intensity was quantified using ImageJ software v1.53e (https://imagej.nih.gov/). Three independent biological replicates were performed.

### Western Blotting Assay.

The *N. benthamiana* leaves (150 mg/sample), *N. tabacum* leaves (150 mg/sample) or rice sheath (50 mg/sample) were harvested and homogenized using RIPA lysis buffer (#P0013B, Beyotime, Beijing, China). The resulting supernatant was added with 6× SDS loading buffer and boiled for 10 min. Proteins were separated by 12.5% SDS-PAGE gel electrophoresis and transferred to PVDF membranes. Then, the membranes were probed with GFP Monoclonal Antibody (GF28R)-HRP (1:5,000, #MA5-15256-HRP, Invitrogen, Carlsbad, CA) or Flag-Tag Antibody (1:10,000 dilution, #MA1-91878, ThermoFisher Scientific) followed by Horseradish Peroxidase-Conjugated Goat Anti-Mouse IgG Antibody (1:10,000, #31430, Thermo Fisher Scientific). Images were acquired by an AI 680 image analyzer (Amersham Pharmacia Biotech, Buckinghamshire, UK). Band intensities in immunoblot analyses were quantified using ImageJ software. Rubisco staining was performed to visualize the sample loading amount. Three independent biological replicates were performed.

### Bioassay in the Tobacco-Whitefly System.

The p35S: miR-7-5P, OsbZIP43-GFP, and OsbZIP43-M-GFP constructs were transiently expressed in *N. tabacum* leaves following a similar procedure to that in *N. benthamiana*. Necrosis effect was observed at 4 d postinfiltration and photographs were obtained using a Canon EOS 80D camera (Canon Inc., Tokyo, Japan).

The host selection experiment was conducted at 48 h postinfiltration, when no necrosis effect was observed. *P. infestans* InF1 was used as a control to indicate cell necrosis. Two leaves with different treatments were placed in a petri dish. Subsequently, 25 newly emerged female whitefly adults were released at the center of the petri dish. The number of insects settling on each leaf was counted at 1, 3, 5, and 24 h after release. At least seven independent biological replicates were conducted.

### Generation of Transgenic Rice Plants.

The *oemir7* and *oebZIP* plants were generated via the custom service of BioRun Biosciences, Wuhan, China. The pBAW(V)HS of pCAMBIA1300 backbone was selected as the vector backbone for both transgenic plants. The diagrams of inserted sequences were displayed in *SI Appendix*, Fig. S13. The pBAW(V)HS-*oemir7* (p35S: miR-7-5P) and pBAW(V)HS-*oebZIP-flag* constructs were introduced into the *A. tumefaciens* strain EHA105. Rice seeds were sterilized with 75% ethanol for 1 min and 50% sodium hypochlorite for 20 min. After washing with sterile water thrice, the sterilized seeds were transferred onto the NBi medium (N6 macroelements, B5 microelements, B5 vitamin, 27.8 mg/L FeSO_4_ · 7H_2_O, 37.3 mg/L Na_2_-EDTA, 500 mg/L proline and glutamic acid, 300 mg/L casein hydrolyte, 2 mg/L 2,4-dichlorophenoxyacetic acid, 100 mg/L inositol, and 30 g/L sucrose) for 20 d at 26 °C for callus induction. The induced calli were incubated with *Agrobacterium* (OD_600_ = 0.2) for 10 min, and then cultured in the NBco medium (NBi medium supplemented with 100 µmol/L acetosyringone, pH 5.5) for 3 d at 20 °C. After washing with sterile water, the calli were transferred onto the NBs medium (NBi medium supplemented with 500 mg/L cephamycin and 30 mg/L hygromycin) for 25 d. Subsequently, the resistant calli were transferred onto the NBr medium (NBi medium supplemented with 0.5 mg/L α-naphthalene acetic acid, 3 mg/L 6-benzylaminopurine, 500 mg/L cephamycin, and 30 mg/L hygromycin) for shoot regeneration. The regenerated shoots were transferred into 1/2× Murashige–Skoog medium for rooting. For *oemir7* plants, successful transformation was confirmed by stem-loop qRT-PCR. For *oebZIP* plants, successful transformation was confirmed by qRT-PCR and western blotting as described above. Two independent homozygous overexpression lines were used for subsequent experiments. The EV transgenic plants, which were generated with the same vector backbone (empty pBAW(V)HS), were used as negative controls.

### Statistical Analysis.

The log-rank test (SPSS Statistics 19, Chicago, IL) was applied to determine the statistical significance of survival distributions. Two-tailed unpaired Student’s *t* test (comparisons between two groups) or one-way ANOVA test followed by Tukey’s multiple comparisons test (comparisons among three groups) was used to analyze the results of honeydew measurement, offspring measurement, and qRT-PCR. The EPG data were first checked for the normality and homogeneity of variance, and those not fitting a normal distribution were log10 transformed before Student’s *t* test, as described in previous studies (52). The Chi-square test was used to analyze the results of host choice experiments. Data were graphed in GraphPad Prism 9.

## Supplementary Material

Appendix 01 (PDF)

## Data Availability

The sequencing data generated in this study have been deposited in the National Genomics Data Center under accession number PRJCA018311 (53). All other data are included in the manuscript and/or *SI Appendix*.
