# Supplementary material for: Cross-kingdom RNA interference mediated by insect salivary microRNAs may suppress plant immunity
Source: Proc Natl Acad Sci U S A. 2024 Apr 8;121(16):e2318783121. doi: 10.1073/pnas.2318783121 (PMC11032475; doi:10.1073/pnas.2318783121)
Supplement: Supplementary file 1 — Appendix 01 (PDF) [file pnas.2318783121.sapp.pdf]

Supplementary Materials for

Cross-kingdom RNA interference mediated by insect salivary microRNAs may suppress plant immunity

Ze-Long Zhang *et al.*

*Corresponding author.
Chuan-Xi Zhang, chxzhang@zju.edu.cn;
Hai-Jian Huang, huanghaijian@nbu.edu.cn

This PDF file includes:

Figs. S1 to S13
Tables S1 to S5

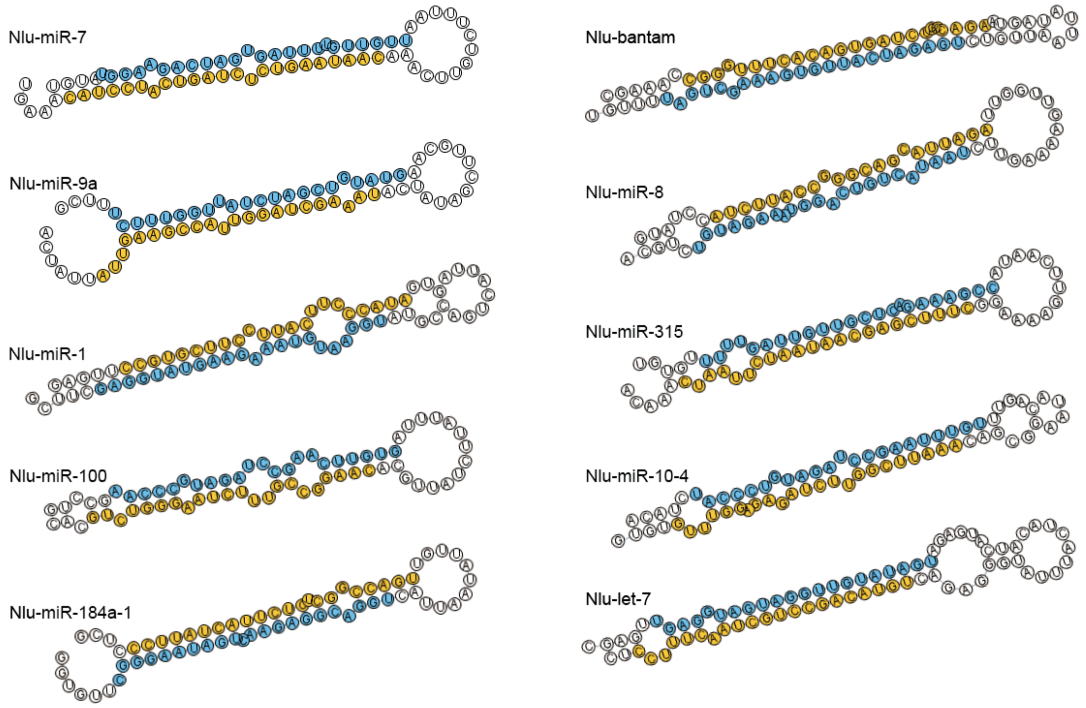

Fig. S1. Secondary structures of miRNA precursors (pre-miRNAs). The mature miRNA located in the 5' end was highlighted by blue, whereas the mature miRNA located in the 3' end was highlighted by yellow. Genome locations of these pre-miRNAs were displayed in Table S1. The sRNAminer software was used to visualize the stem-loop structure.

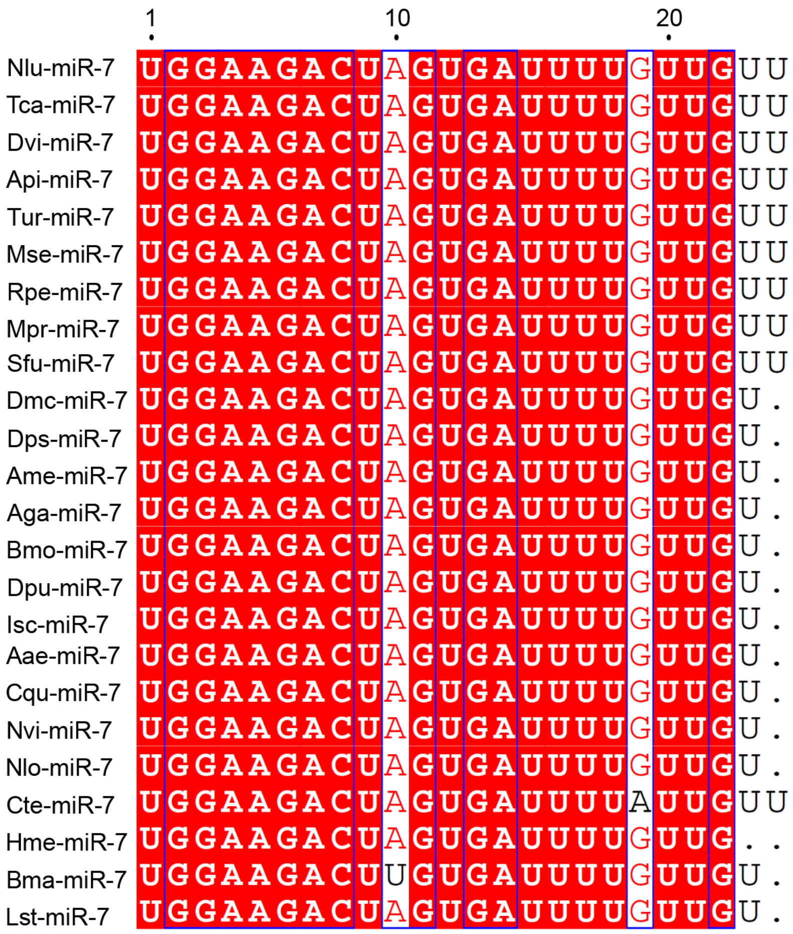

Fig. S2. Sequence alignment of insect miR-7s. The miR-7 from *Nilaparvata lugens* (Nlu), *Tribolium castaneum* (Tca), *Drosophila virilis* (Dvi) , *Acyrthosiphon pisum* (Api), *Tetranychus urticae* (Tur), *Manduca sexta* (Mse), *Riptortus pedestris* (Rpe), *Myzus persicae* (Mpe), *Sogatella furcifera* (Sfu), *Drosophila mclanogaster* (Dmc), *Drosophila pseudoobscura* (Dps), *Apis mellifera* (Ame), *Anopheles gambiae* (Aga), *Bombyx mori* (Bmo), *Daphnia pulex* (Dpu) , *Ixodes scapularis* (Isc), *Aedes aegypti* (Aae), *Culex quinqucfasciatus* (Cqu), *Nasonia vitripennis* (Nvi), *Nasonia longicornis* (Nlo), *Capitella teleta* (Cte), *Heliconius melpomene* (Hme), *Brugia malayi* (Bma), *Laodelphax striatellus* (Lst) were aligned using Espript software (https://espript.ibcp.fr/ESPript/ESPript/). Red shading indicates the conserved regions of miR-7.

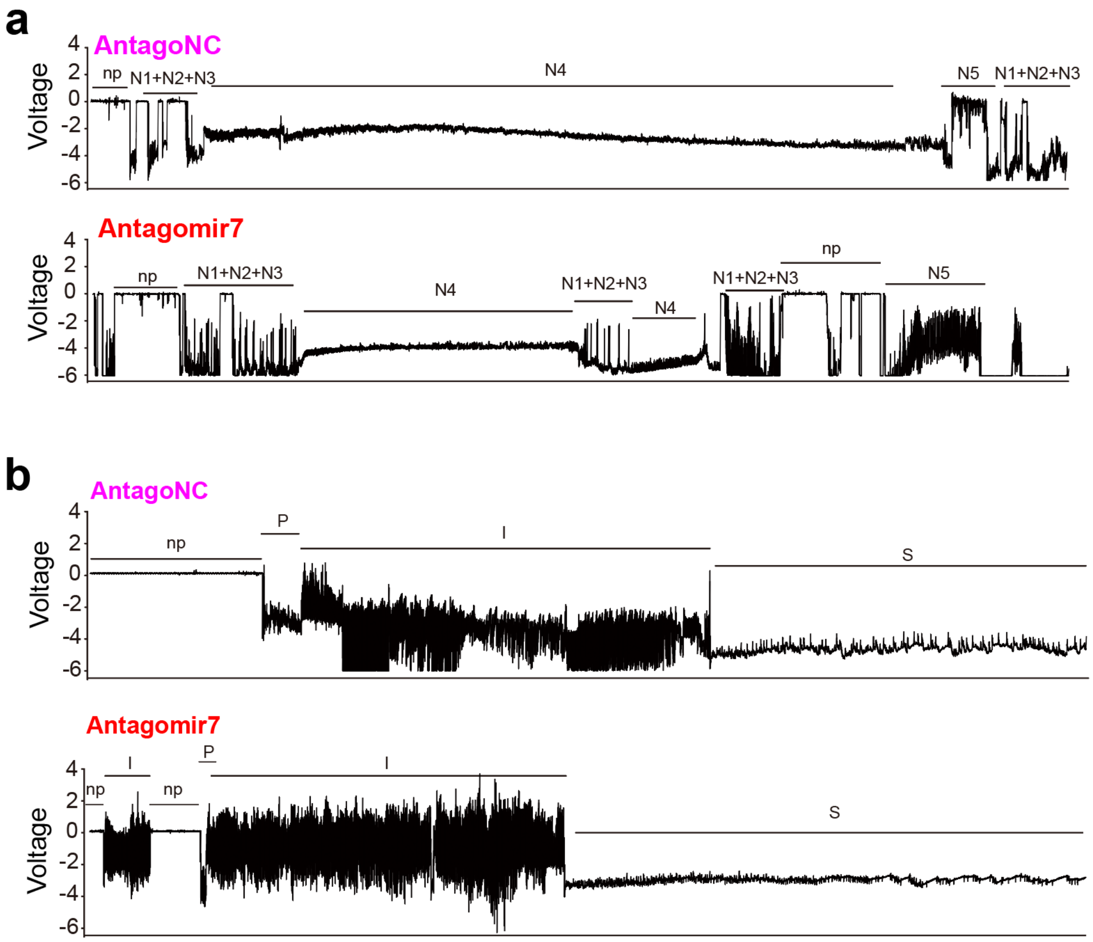

Fig. S3. Typical EPG waveforms of antagoNC- and antagomir7-treated *Nilaparvata lugens*. EPG waveforms over 1 h for antagoNC- and antagomir7-treated *N. lugens* on rice plants (**a**) and artificial diets (**b**) were displayed. Insect feeding behavior on rice plants can be classified into nonpenetration (np), pathway duration (N1+N2+N3), phloem sap ingestion (N4), and xylem sap ingestion (N5) phases (**a**). The insect feeding behavior on artificial diets can be classified into np, penetration initiation (P), salivation and stylets movement (S), and ingestion (I) phases (**b**).

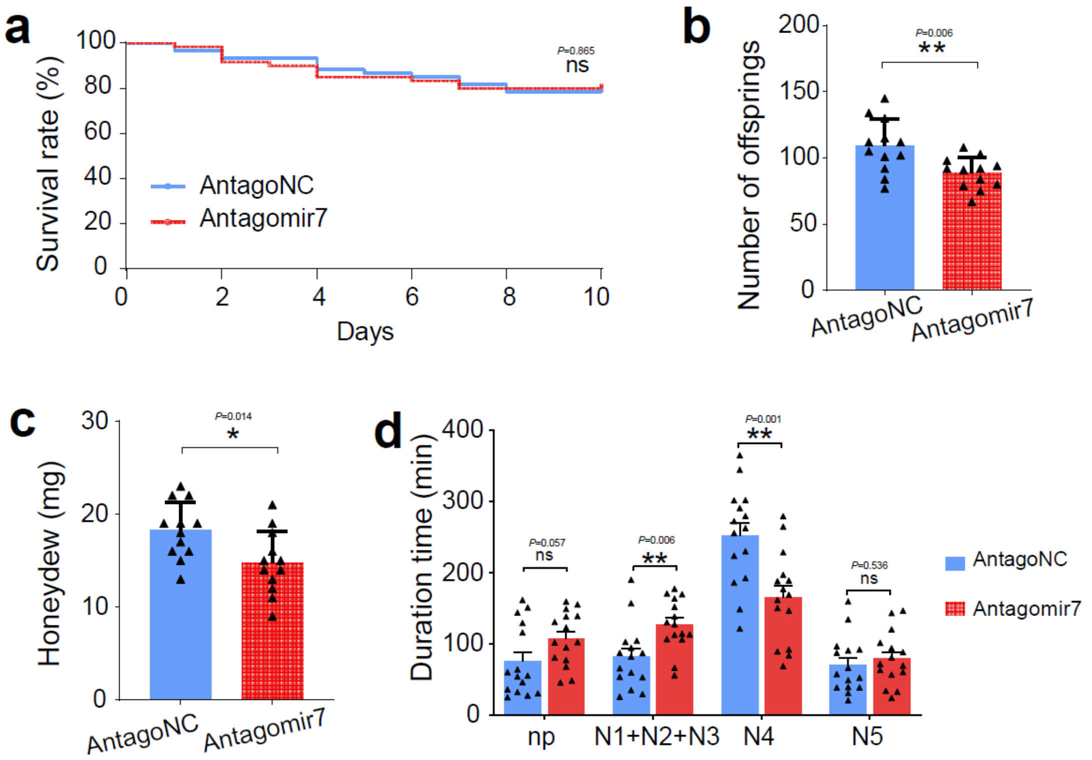

Fig. S4. Effects of miR-7-5P suppression on *Nilaparvata lugens* feeding on rice variety cv. Xiushui. *N. lugens* were treated with antagomir7 or antagoNC and reared on the rice variety cv. Xiushui. Insect survivorship **(a)**, reproduction **(b)**, honeydew excretion **(c)**, and feeding behavior **(d)** were tested**.** Insect feeding behavior on rice plants can be classified into nonpenetration (np), pathway duration (N1+N2+N3), phloem sap ingestion (N4), and xylem sap ingestion (N5) phases. For survival analysis, n= 60 individuals in each treatment. For reproduction, honeydew and EPG analyses, n=12, n=12, and n= 15 independent biological replicates, respectively. Differences in reproduction, honeydew excretion and EPG recording were determined by two-tailed unpaired Student's *t*-test. ***P<0.001; *P<0.05; ns, not significant. Data in (**a**), (**c**), and (**d**) are presented as mean ± SEM.

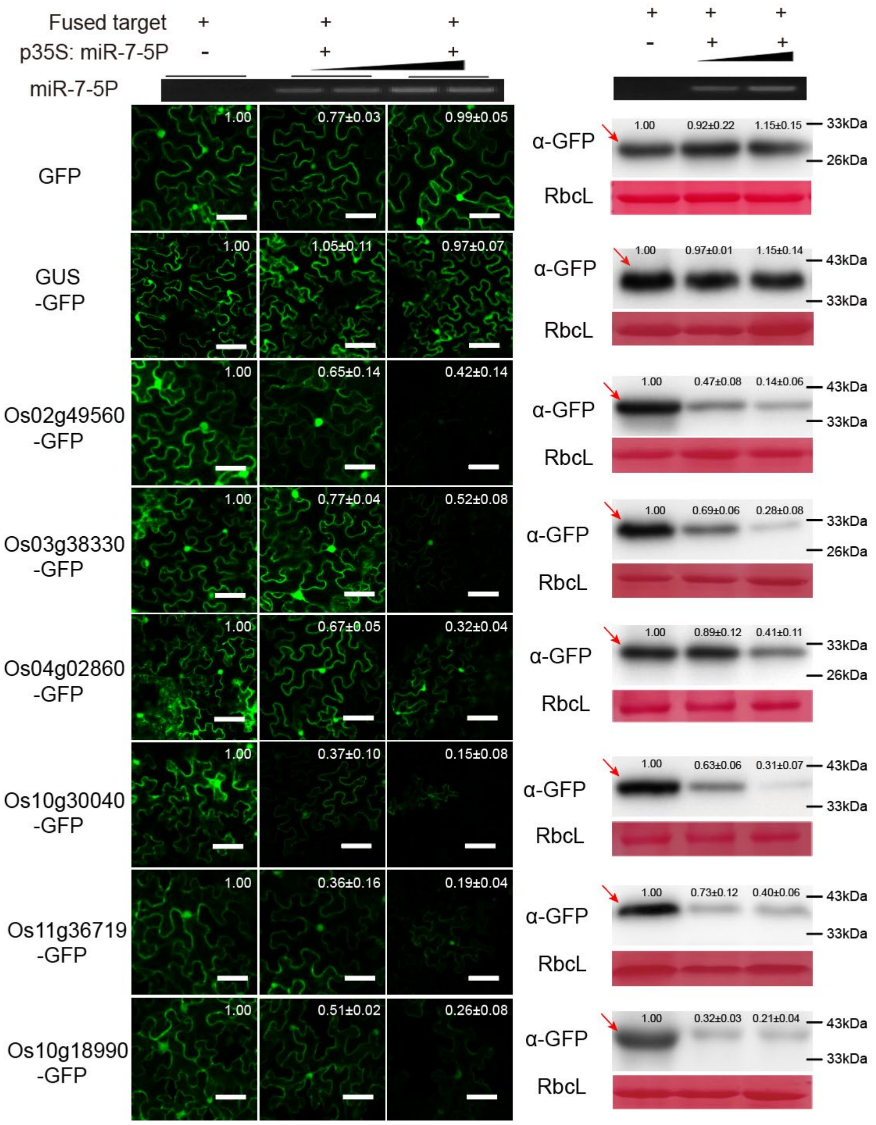

Fig. S5. Validation of the interaction between miR-7-5P and target sequences. DNA fragments approximately 100 bp upstream and downstream of the target sites were ligated to the 3' end of the *GFP* gene. The recombinant plasmid was co-infiltrated into *Nicotiana benthamiana* leaves along with varying concentration of p35S: miR-7-5P. The miR-7-5P level in leaves was determined by stem-loop qRT-PCR. Confocal microscopy (left) and Western-blotting (right) were performed. Rubisco staining (RbcL) was conducted to visualize the amount of sample loading. Vectors encoding GFP protein alone or recombinant β-glucuronidase (GUS)-GFP protein were served as negative controls. Bar = 50 μm. The target genes and their corresponding codes are as follows: Os02g49560 (OsbZIP43), Os03g38330 (NBS-LRR-like resistance protein), Os04g02860 (disease resistance protein RPM1), Os10g30040 (BTB/POZ domain containing protein), Os11g36719 (lipoxygenase), and Os10g18990 (receptor kinase 2). The target band was indicated by red arrows. Three independent biological replicates were performed, and the representative fluorescence and Western-blotting images were displayed. The intensity/density value from three biological replicates were calculated using ImageJ, and the mean value in the controls were set as 1.0. Data are presented as mean ± SEM (n= 3 independent biological replicates). The small triangle indicates the different concentration (OD_{600}= 0.3 and 1.0) of *Agrobacterium* harboring p35S: miR-7-5P.

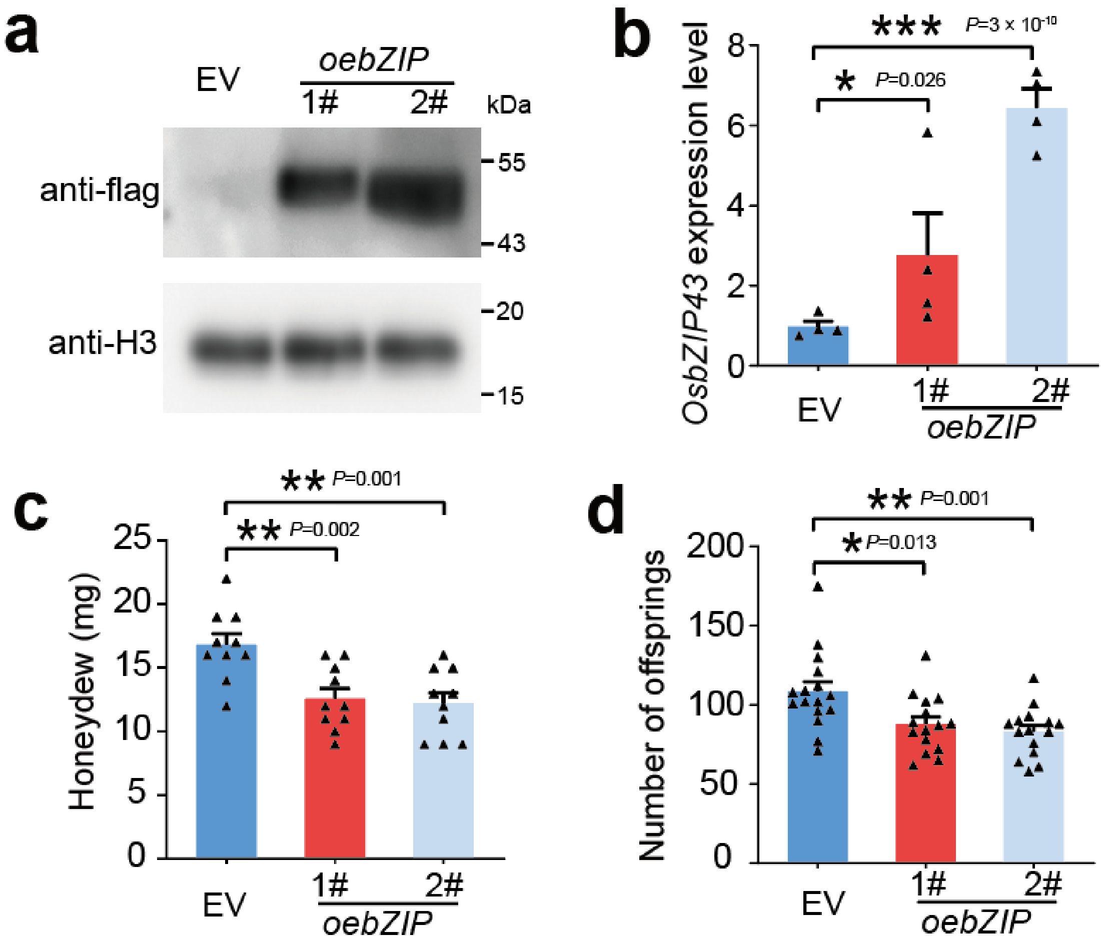

Fig. S6. Effects of OsbZIP43 overexpression on rice plants. (a, b) Detection of OsbZIP43 in transgenic plant *oebZIP*#1 and *oebZIP*#2 using Western-blotting **(a)** and qRT-PCR assays **(b)**. The empty vector (EV) transgenic plants served as a control. The full open reading frames of OsbZIP43 with a flag-tag at the C-terminus was overexpressed. The flag-tag antibody was used to detect the presence of OsbZIP43-flag protein. Histone H3 antibody was used to visualize the amount of sample loading. **(c, d)** Effects of OsbZIP43 overexpression on insect honeydew excretion **(c)** and reproduction **(d)**. *Nilaparvata lugens* were allowed to feed on indicated rice plants, respectively. For honeydew analysis, n= 10 independent biological replicates. For reproduction analysis, n= 16, 15, and 15 independent biological replicates on EV, *oebZIP*#1, and *oebZIP*#2, respectively. *P*-values were determined by two-tailed unpaired Student's *t* test. ***$P < 0.001$; **$P < 0.01$; *$P<0.05$. Data are presented as mean ± SEM.

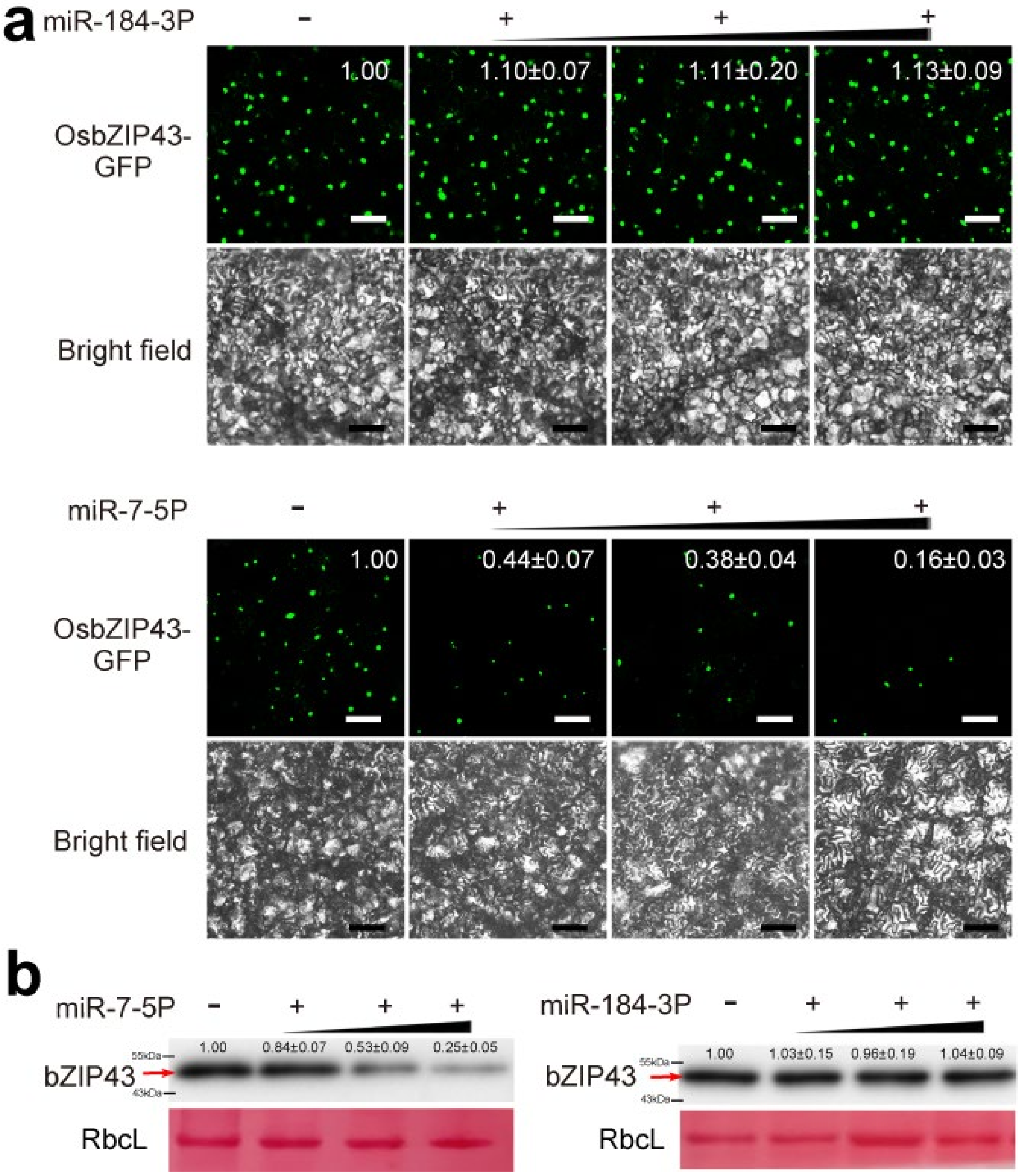

Fig. S7. Suppression of OsbZIP43 expression by miR-7-5P, but not miR-184-3P. At 48-h after co-infiltration, green fluorescence of the OsbZIP43-GFP was observed in *Nicotiana benthamiana* leaves **(a)**, and the protein level of GFP-fused proteins was determined by a Western-blotting assay **(b)**. Bar = 100 µm. miR-184-3P was used as a negative control. Rubisco staining (RbcL) was conducted to visualize the amount of sample loading. Three independent biological replicates were performed, and the representative fluorescence and Western-blotting images were displayed. The intensity/density value from three biological replicates were calculated using ImageJ, and the mean value in the controls were set as 1.0. Data are presented as mean ± SEM (n= 3 independent biological replicates). The small triangle indicates the different concentration (OD_{600}=0.05, 0.3, and 1.0) of *Agrobacterium* harboring p35S: miR-7-5P or p35S: miR-184-3P.

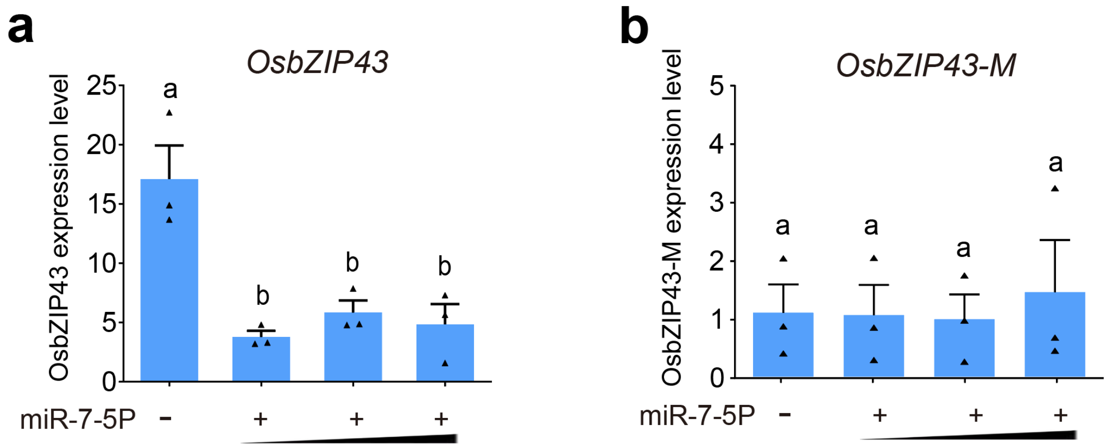

Fig. S8. Effects of miR-7-5P on the transcript level of *OsbZIP43* and *OsbZIP43-M*. Co-infiltration of miR-7-5P was performed with OsbZIP43 and OsbZIP43-M, respectively. The relative transcript levels of *OsbZIP43* **(a)** and *OsbZIP43-M* **(b)** were determined using qRT-PCR. Different lowercase letters indicate statistically significant differences at $P < 0.05$ level according to one-way ANOVA test followed by Tukey's multiple comparisons test. Data are presented as mean ± SEM (n= 3 independent biological replicates). The small triangles below the figure indicate the different concentration (OD_{600}=0.05, 0.3, and 1.0) of *Agrobacterium* harboring p35S: miR-7-5P.

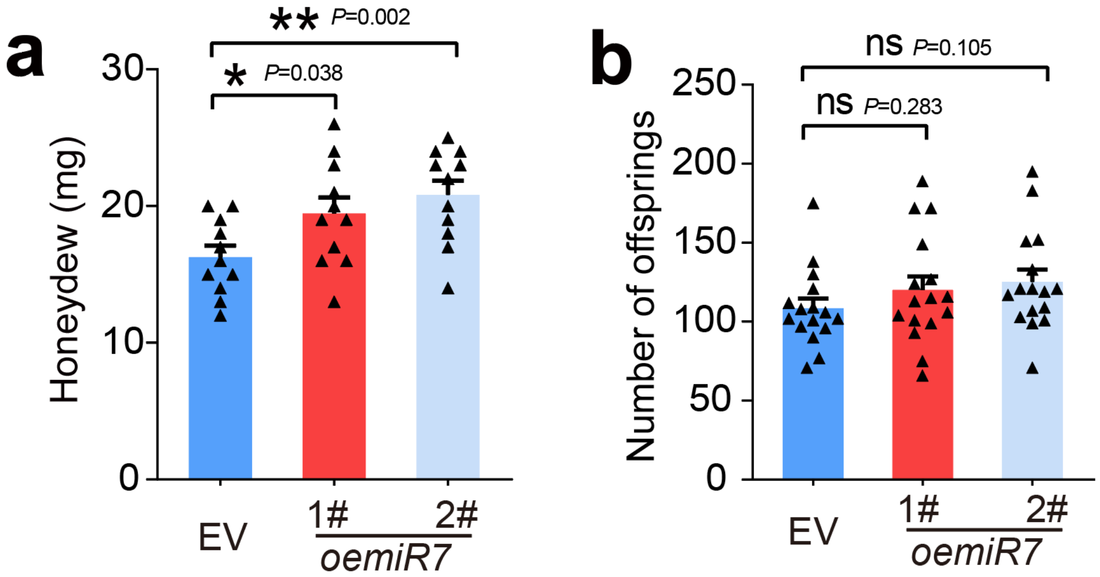

Fig. S9. Effects of miR-7-5P overexpression on insects. *Nilaparvata lugens* were allowed to feed on transgenic plant *oemir7*#1 and *oemir7*#2. Honeydew excretion **(a)** and reproduction **(b)** were determined. The empty vector (EV) transgenic plants served as a control. n= 11 and 16 independent biological replicates in honeydew and reproduction analyses, respectively. *P*-values were determined by two-tailed unpaired Student's *t* test. ***P* < 0.01; **P*<0.05; ns, not significant. Data are presented as mean ± SEM.

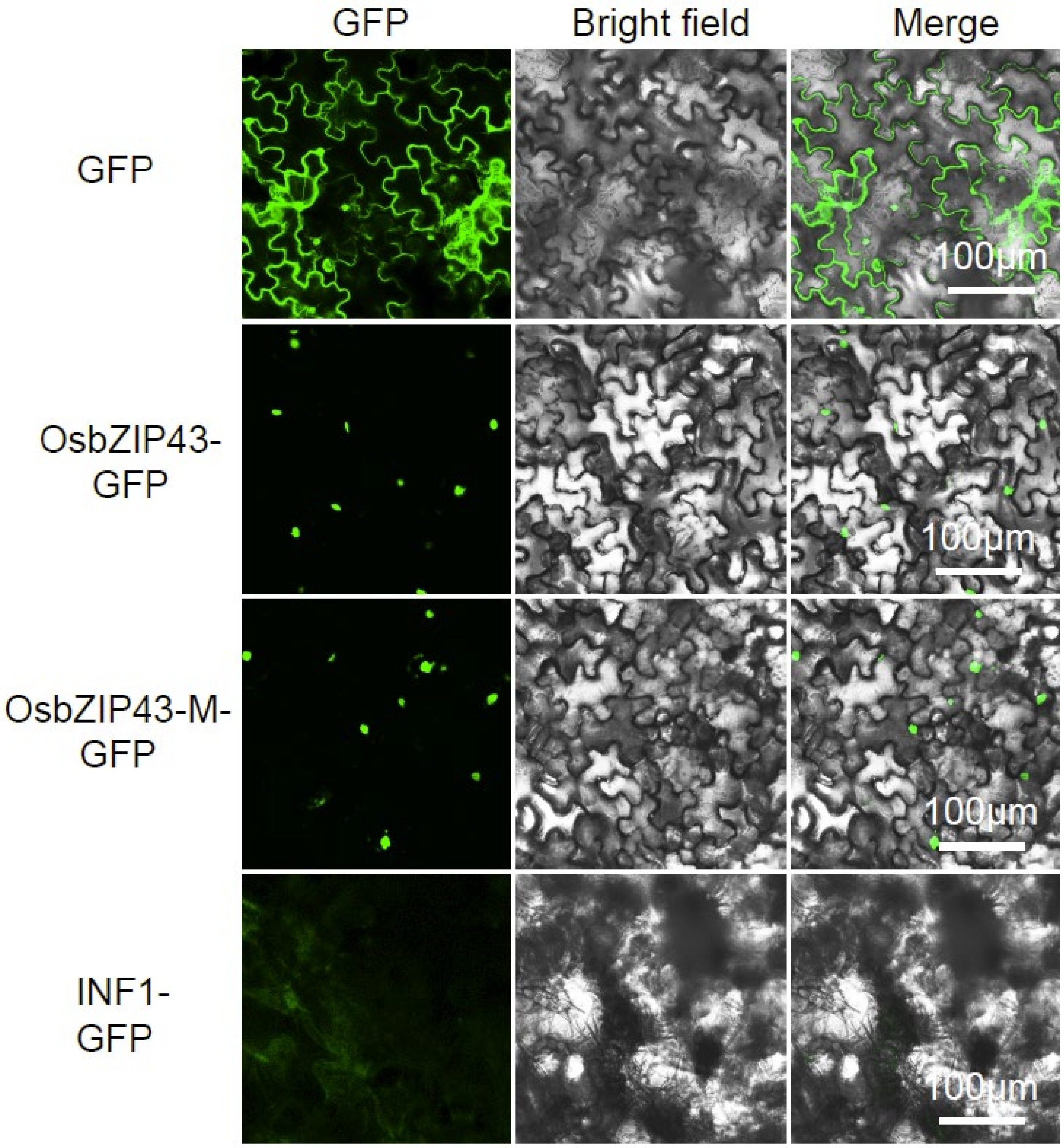

Fig. S10. Effects of OsbZIP43-GFP and OsbZIP43-M-GFP overexpression on *Nicotiana tabacum*. The OsbZIP43-GFP, OsbZIP43-M-GFP, and GFP were transiently expressed in *N. tabacum*, respectively. At 48 h post-infiltration, cell morphology and fluorescence signal derived from GFP expression were observed. No necrosis effect was detected in GFP-, OsbZIP43-GFP- or OsbZIP43-M-GFP-expressing leaves at 48 h post-infiltration. In contrast, leaves infiltrated with InF1, a necrosis-inducing protein from *Phytophthora infestans*, exhibited significant cell morphological changes.

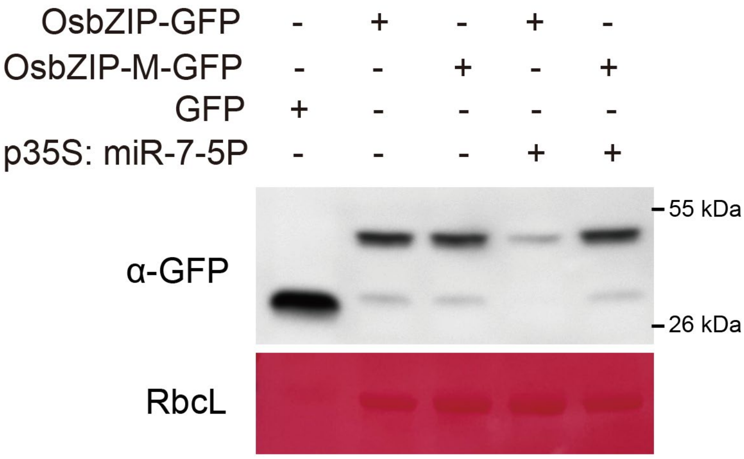

Fig. S11. Protein levels of infiltrated *Nicotiana tabacum* leaves. The OsbZIP43-GFP, OsbZIP43-M-GFP, GFP, and miR-7-5P were transiently expressed in *N. tabacum*. At 48 h post-infiltration, the protein levels of OsbZIP43-GFP, OsbZIP43-M-GFP, and GFP were determined by Western-blotting assay using a GFP Monoclonal Antibody. Rubisco staining (RbcL) was conducted to visualize the sample loading amount.

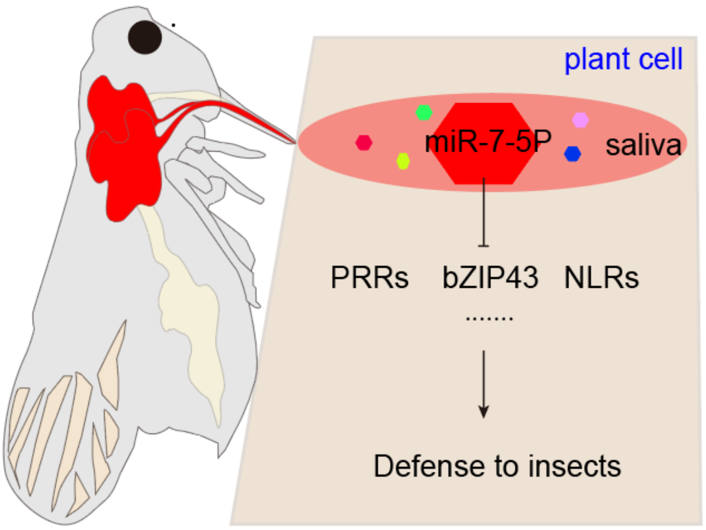

Fig. S12. The proposed model for the cross-kingdom RNA interference between *Nilaparvata lugens* and rice plants. During the feeding process, *N. lugens* secreted a few salivary miRNAs into rice plants, including the miR-7-5p, which is specifically expressed in the salivary glands. The miR-7-5P enhances insect feeding on rice plants by suppressing multiple immune-associated genes in host plants, such as the bZIP transcription factor 43 (bZIP43), pattern-recognition receptors (PRRs), and nucleotide-binding domain, leucine-rich repeat domain-containing receptors (NLRs).

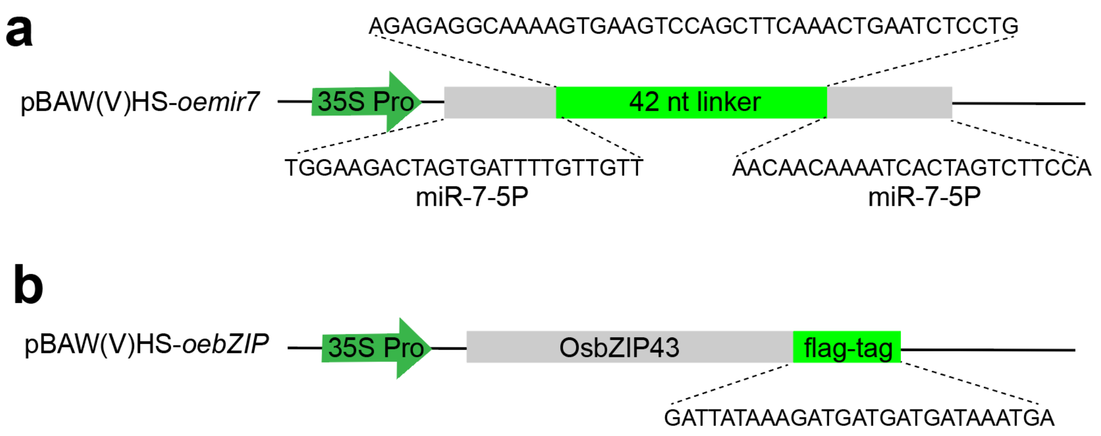

Fig. S13. The diagram of inserted sequences in constructing vectors for transgenic rice plants. The pBAW(V)HS of pCAMBIA1300 backbone was selected for vector construction. For *oemir7* plants **(a)**, the looped sequence was inserted after CaMV 35S promoter (35S Pro), while for *oebZIP* plants **(b)**, the full open reading frames of OsbZIP43 with a flag-tag at the C-terminus was inserted after 35S Pro.

Table S1. Identification of insect-derived miRNA in saliva

miRNA	Sequence	Loci (*Nilaparvata lugens*)	Loci (*Oryza sativa*) [1]	Abundance [2]
Nlu-miR-100-5P	AACCCGTAGATCCGAACTTGTG	chrX+:76470349-76470415	ND	606.0
Nlu-miR-7-5P	TGGAAGACTAGTGATTTTGTTGTT	chr2+:26963202-26963271	ND	512.6
Nlu-miR-184-3P	TGGACGGAGAACTGATAAGGGC	chr10+:37774442-37774507	ND	148.8
Nlu-let-7-5P	TGAGGTAGTAGGTTGTATAGT	chrX+:76472809-76472883	ND	126.9
Nlu-miR-10-4-5P	TACCCTGTAGATCCGAATTTGT	chr8-:16632161-16632229	ND	91.7
Nlu-miR-9a-5P	TCTTTGGTTATCTAGCTGTATG	chr2+:90035669-90035736	ND	80.2
Nlu-miR-1-3P	TGGAATGTAAAGAAGTATGGAG	chr3+:10186832-10186901	ND	60.0
Nlu-miR-8-3P	TAATACTGTCAGGTAAAGATGT	chr14+:8123571-8123639	ND	53.1
Nlu-miR-315-5P	TTTTGATTGTTGCTCAGAAAGCC	chrX-:31029248-31029316	ND	31.5
Nlu-Bantam-3P	TGAGATCATTGTGAAAGCTGAT	chr5+:22533234-22533305	ND	19.7
Nlu-miR-8-5P	CATCTTACCGGGCAGCATTAGA	chr14+:8123571-8123639	ND	12.0

[1] ND, not detected

[2] The abundance of miRNAs was displayed as transcripts per million (TPM)

Table S2. Identification of insect-derived miRNA in infested plants[1]

miRNA	Non-infested R1	Non-infested R2	Non-infested R3	Non-infested R4	Infested R1	Infested R2	Infested R3	Infested R4
Nlu-miR-100-5P	3.8	4.9	2.7	3.0	86.9	10.7	29.9	46.9
Nlu-miR-7-5P	0	0	0	0	42.1	23.9	16.3	15.2
Nlu-miR-184-3P	0	1.6	0	0	20.2	27.2	2.3	7.6
Nlu-miR-9a-5P	0	0	0	0	94.0	8.0	7.3	6.9
Nlu-miR-1-3P	0	0	0	0	7.3	29.4	4.2	0.8

[1] The abundance of miRNAs was displayed as transcripts per million (TPM)

Table S3. Expression patterns of miRNA in different tissues[1]

miRNA	SG	gut	FB	Te	Ov
Nlu-miR-100-5P	427,599	230,448	282,506	86,769	175,095
Nlu-miR-7-5P	103,859	1,628	5,654	14,776	15,003
Nlu-miR-184-3P	130,104	48,736	81,300	8,986	14,845
Nlu-miR-9a-5P	87,332	41,011	93,158	5,150	18,130
Nlu-miR-1-3P	122,283	52,058	55,537	8,878	8,218
Total sRNAs	26,014,059	20,299,578	225,157,32	279,860,28	243,139,35

[1] The expression of miRNAs in salivary glands (SG), guts, fat bodies (Fb), testes (Te), and ovaries (Ov) were quantified by sRNA sequencing. The read counts of specific miRNAs and total sRNAs were displayed.

Table S4. Targets of miR-7-5P in rice plant.

**Target_Gene**	**psRNATarget** (Expectation)	**miRanda** (Total Energy/) kcal/mol	**RNAhybrid** (Total Energy) kcal/mol	**Description**
Os02g49560.1	3	-34.9	-27.6	bZIP transcription factor domain containing protein
Os09g31025.1	3	-23.5	-26.5	EF hand family protein
Os08g23680.3	3.5	-45.4	-27.7	FHA domain containing protein, putative
Os03g38330.1	3.5	-42.1	-31.0	NBS-LRR-like resistance protein
Os08g43460.1	3.5	-27.0	-29.6	Expressed protein
Os07g17280.1	3.5	-25.1	-29.6	Ser/Thr protein phosphatase family protein, putative
Os10g42170.1	3.5	-24.0	-30.0	GTPase of unknown function domain containing protein, putative
Os07g03150.1	3.5	-24.6	-27.9	H-BTB5 - Bric-a-Brac, Tramtrack, Broad Complex BTB domain with H family conserved sequence
Os11g36790.1	3.5	-23.1	-28.4	OsFBO6 - F-box and other domain containing protein
Os07g04530.1	4	-39.9	-30.2	GYF domain containing protein, putative
Os01g44360.1	4	-30.8	-24.0	CBS domain containing membrane protein, putative
Os04g02860.1	4	-43.2	-24.8	Disease resistance protein RPM1, putative
Os03g33610.1	4	-22.9	-27.4	Retrotransposon, putative, centromere-specific
Os10g30040.1	4	-29.1	-33.7	BTB/POZ domain containing protein, putative
Os03g16070.1	4	-21.6	-25.0	Expressed protein
Os10g04480.1	4	-21.2	-27.5	Expressed protein
Os04g03180.1	4	-14.9	-27.2	Disease resistance protein, putative
Os04g49530.1	4	-19.2	-23.6	Expressed protein
Os02g52490.1	4	-22.9	-25.1	Expressed protein
Os05g28170.1	4.5	-71.3	-26.3	Expressed protein

Os02g48360.1	4.5	-55.1	-28.0	Pyrophosphate--fructose 6-phosphate 1-phosphotransferase subunit alpha, putative
Os11g36719.1	4.5	-59.7	-29.9	Lipoxygenase, putative
Os07g03025.1	4.5	-41.5	-30.7	Expressed protein
Os02g38020.1	4.5	-50.7	-31.4	Inorganic phosphate transporter 2-1, chloroplast precursor, putative
Os07g25390.1	4.5	-43.7	-28.1	DRD1, putative
Os02g45650.1	4.5	-48.2	-28.3	Peptidase, putative
Os12g40070.1	4.5	-44.3	-34.1	B3 DNA binding domain containing protein
Os03g02762.1	4.5	-37.6	-26.1	PPR repeat domain containing protein, putative
Os01g41280.1	4.5	-31.5	-34.0	OsFBD4 - F-box and FBD domain containing protein
Os05g34820.1	4.5	-40.1	-23.6	Expressed protein
Os01g56780.1	4.5	-19.9	-26.9	Plus-3 domain containing protein
Os01g05850.1	4.5	-26.0	-29.0	Expressed protein
Os09g29750.1	4.5	-19.6	-26.7	ZOS9-15 - C2H2 zinc finger protein
Os02g27020.1	4.5	-19.4	-23.8	Expressed protein
Os01g03810.1	4.5	-25.2	-28.5	Expressed protein
Os02g07910.1	4.5	-23.1	-26.1	Fiber protein Fb15, putative
Os05g33270.1	4.5	-15.4	-26.0	GDSL-like lipase/acylhydrolase, putative
Os02g34800.1	4.5	-24.0	-28.2	Leucine-rich repeat receptor protein kinase EXS precursor, putative
Os04g03850.1	4.5	-23.1	-26.9	OsSub39 - Putative Subtilisin homologue
Os02g52230.1	4.5	-23.1	-27.1	Coronatine-insensitive protein 1, putative
Os03g27250.1	4.5	-18.3	-24.4	F-box and other domain-containing protein
Os01g67290.1	5	-78.2	-29.8	Cyclin-related protein, putative
Os08g17370.1	5	-40.2	-29.6	Transmembrane 9 superfamily member, putative
Os10g18990.1	5	-40.7	-27.8	Receptor kinase 2, putative
Os05g43240.1	5	-38.4	-24.2	Jacalin-like lectin domain containing protein

Os03g61440.1	5	-26.0	-29.7	Uncharacterized Cys-rich domain containing protein, putative
Os11g32590.1	5	-17.4	-27.6	Transposon protein, putative, Mariner sub-class
Os05g08990.1	5	-22.0	-26.5	Glutaminyl-tRNA synthetase, putative
Os12g37760.1	5	-24.6	-27.7	RGH1A, putative
Os03g48840.1	5	-17.7	-31.0	Ribosomal L18p/L5e family protein, putative
Os02g02610.1	5	-25.6	-29.6	Transposon protein, putative, unclassified
Os09g02650.1	5	-21.0	-29.6	Motor domain

Table S5. Primers used in this study

Gene	Forward（5’-3’）	Reverse（5’-3’）
**Primers used in plasmid construction**		
OsbZIP43	ATGTACCCTGCTGAGATT	TCAATTGTCAGGACCCAT
GFP-OsbZIP43	ACGAGCTGTACAAGGGTACCATGTACCCTGCTGAGATT	GCGGACTCTAGTTCATCTAGATCAATTGTCAGGACCCAT
GFP-fOsbZIP43	ACGAGCTGTACAAGGGTACCATGTACCCTGCTGAGATT	GCGGACTCTAGTTCATCTAGATCAACTGCGTTGATAGTCA
GFP-36719	ACGAGCTGTACAAGGGTACCATGGTCGAGGGCCTCACCGTC	GCGGACTCTAGTTCATCTAGATCACGTGTAGACCTCGCC
GFP-18990	ACGAGCTGTACAAGGGTACCATGTCTGACTCTTACTCTTTC	GCGGACTCTAGTTCATCTAGATCATGGCACAATGAACAT
GFP-38330	ACGAGCTGTACAAGGGTACCATGACCTGGAGGTATAAGAA	GCGGACTCTAGTTCATCTAGATTAAAGACCAAAGTAGTGAG
GFP-30150	ACGAGCTGTACAAGGGTACCATGGTCACCAGAGGATGCTG	GCGGACTCTAGTTCATCTAGATTAAAATGTTAGTAACAATCA
GFP-02860	ACGAGCTGTACAAGGGTACCATGAGTTCACACAATCCAACA	GCGGACTCTAGTTCATCTAGATTATGCCTGTAGCTTAAACCT
GFP-30040	ACGAGCTGTACAAGGGTACCATGGCGACAACGACGGCG	GCGGACTCTAGTTCATCTAGACTAGACATCTGCATTTT
OsbZIP43-flag	CGACGACAAGACCGTCACCATGTACCCTGCTGAGATT	GAGGAGAAGAGCCGTCGTCAATTGTCAGGACCCAT
GFP-Os-M-bZIP43	ATGTACCCTGCTGAGATTGCGCACACAAAGTACTTGTC	GCGGACTCTAGTTCATCTAGATCAATTGTCAGGACCCAT
**Primers used in qRT-PCR**		
qRT-OsbZIP43	GCAAGACTGCGTTGATAG	AGATACAAATGACTTCCTCCTC
qRT-OsbZIP43	CCTGCTGGAGTTCGTGAC	GTTATACTGGAGGAGGAAGTCAT
Os-Actin	ACCACTTCGACCGCCACTACT	ACGCCTAAGCCTGCTGGTT
Nb-Actin	GGAGAAGTTGGCTTACATTG	TCATTGATGGTTGGAACAGA
**Primers used in cDNA synthesis**		
Nlu-miR-184a	CTCAACTGGTGTCGTGGAGTCGGCAATTCAGTTGAGCGCCCTTAA	
Nlu-miR-7-5P	CTCAACTGGTGTCGTGGAGTCGGCAATTCAGTTGAGCAACAACAA	
Nlu-miR-9a-5p	CTCAACTGGTGTCGTGGAGTCGGCAATTCAGTTGAGCCATACAG	

miR-1-3p	CTCAACTGGTGTCGTGGAGTCGGCAATTCAGTTGAGCCTCCATAC	
bantam-3p	CTCAACTGGTGTCGTGGAGTCGGCAATTCAGTTGAGCATCAGCTT	
miR-100-5p	CTCAACTGGTGTCGTGGAGTCGGCAATTCAGTTGAGCCACAAGTT	
Rp-miR-7	CTCAACTGGTGTCGTGGAGTCGGCAATTCAGTTGAGCAACAACAA	
Dme/Ls-miR7	CTCAACTGGTGTCGTGGAGTCGGCAATTCAGTTGAGCACAACAAA	
**Primers used in stem-loop qRT-PCR**		
miR-184a	CTCAACTGGTGTCGTGGA	TCGGCAGGTGGACGGAGAAC
miR-7-5p	CTCAACTGGTGTCGTGGA	TCGGCAGGTGGAAGACTAGT
miR-9a-5p	CTCAACTGGTGTCGTGGA	TCGGCAGGTCTTTGGTTAT
miR-1-3p	CTCAACTGGTGTCGTGGA	TCGGCAGGTGGAATGTAAAG
bantam-3p	CTCAACTGGTGTCGTGGA	TCGGCAGGTGAGATCATTGT
miR-100-5p	CTCAACTGGTGTCGTGGA	TCGGCAGGAACCCGTAGATC
Sf/Ls/Nl-U6	CTCAACTGGTGTCGTGGAGTCGGCAATTCAGTTGAGCAAAAAATGTG	GTGCAGGGTCCGAGGT
Os-U6	CGATAAAATTGGAACGATACAGA	ATTTGGACCATTTCTCGATTTGT
Mp/Sf/Rp/Nl-miR-7	CTCAACTGGTGTCGTGGAGTCGGCAATTCAGTTGAGCAACAACAA	GTGCAGGGTCCGAGGT
Dme/Ls-miR7	CTCAACTGGTGTCGTGGAGTCGGCAATTCAGTTGAGCACAACAAA	GTGCAGGGTCCGAGGT
